# Supplementary material for: Determining the minimal important differences in the International Prostate Symptom Score and Overactive Bladder Questionnaire: results from an observational cohort study in Dutch primary care
Source: BMJ Open. 2019 Dec 23;9(12):e032795. doi: 10.1136/bmjopen-2019-032795 (PMC7008409; doi:10.1136/bmjopen-2019-032795)
Supplement: Supplementary data [file bmjopen-2019-032795supp001.pdf]

INTERNATIONAL-PROSTATE SYMPTOM SCORE (IPSS)

	**Not at all**	**Less than 1 time in 5**	**Less than half the time**	**About half the time**	**More than half the time**	**Almost always**
1. Over the past 4 weeks, how often have you had a sensation of not emptying your bladder completely after you finished urinating?	0	1	2	3	4	5
2. Over the past 4 weeks, how often have you had to urinate again less than two hours after you finished urinating?	0	l	2	3	4	5
3. Over the past 4 weeks, how often have you found you stopped and started again several times when you urinated?	0	1	2	3	4	5
4. Over the past 4 weeks, how often have you found it difficult to postpone urination?	0	1	2	3	4	5
5 Over the past 4 weeks, how often has your urinary stream been weaker than usual?	0	1	2	3	4	5
6 Over the past 4 weeks, how often have you had to push or strain to begin urination?	0	1	2	3	4	5
	None	1 time	2 times	3 times	4 times	5 or more times
7. Over the past 4 weeks, how many times, in general, did you get up to urinate from the time you went to bed at night until the time you got up in the morning?	0	1	2	3	4	5

IPSS Quality of life question

	Delighted	Pleased	Mostly satisfied	Mixed - neither satisfied nor dissatisfied	Mostly dissatisfied	Unhappy	Terrible
If you were to spend the rest of your life with your urinary condition just the way it is now, how would you feel about that?	0	1	2	3	4	5	6

OAB-q-SF

This questionnaire asks about how much you have been bothered by selected bladder symptoms during the past 4 weeks. Please place a or X in the box that best describes the extent to which you were bothered by each symptom during the past 4 weeks. There are no right or wrong answers. Please be sure to answer every question.

During the past 4 weeks, how bothered were you by…

	Not at all	A little bit	Some-what	Quite a bit	A great deal	A very great deal
1. An uncomfortable urge to urinate?	☐	☐	☐	☐	☐	☐
2. A sudden urge to urinate with little or no warning?	☐	☐	☐	☐	☐	☐
3. Accidental loss of small amounts of urine?	☐	☐	☐	☐	☐	☐
4. Nighttime urination?	☐	☐	☐	☐	☐	☐
5. Waking up at night because you had to urinate?	☐	☐	☐	☐	☐	☐
6. Urine loss associated with a strong desire to urinate?	☐	☐	☐	☐	☐	☐

Patient Global Impression of Improvement, PGI-I

Have your symptoms changed since the start of the medication (the moment you completed the previous questionnaire)?

Very much better	Much better	A little better	No change	A little worse	Much worse	Very much worse
☐	☐	☐	☐	☐	☐	☐
