# Supplementary material for: Determining the minimal important differences in the International Prostate Symptom Score and Overactive Bladder Questionnaire: results from an observational cohort study in Dutch primary care
Source: BMJ Open. 2019 Dec 23;9(12):e032795. doi: 10.1136/bmjopen-2019-032795 (PMC7008409; doi:10.1136/bmjopen-2019-032795)
Supplement: Author's manuscript [file bmjopen-2019-032795.draft_revisions.pdf]

BMJ Open is committed to open peer review. As part of this commitment we make the peer review history of every article we publish publicly available.

When an article is published we post the peer reviewers' comments and the authors' responses online. We also post the versions of the paper that were used during peer review. These are the versions that the peer review comments apply to.

The versions of the paper that follow are the versions that were submitted during the peer review process. They are not the versions of record or the final published versions. They should not be cited or distributed as the published version of this manuscript.

BMJ Open is an open access journal and the full, final, typeset and author-corrected version of record of the manuscript is available on our site with no access controls, subscription charges or pay-per-view fees (http://bmjopen.bmj.com).

If you have any questions on BMJ Open's open peer review process please email info.bmjopen@bmj.com

BMJ Open

Minimal important differences in the International Prostate Symptom Score and Overactive Bladder Questionnaire in a primary care population

Journal:	*BMJ Open*
Manuscript ID	bmjopen-2019-032795
Article Type:	Research
Date Submitted by the Author:	06-Jul-2019

Complete List of Authors:	Blanker, Marco; University of Groningen, University Medical Center Groningen, department of General Practice and Elderly Care Medicine<br>Alma, Harma; University of Groningen, University Medical Center Groningen, Department of General Practice and Elderly Care Medicine; University of Groningen, University Medical Center Groningen, Groningen Research Institute for Asthma and COPD (GRIAC)<br>Devji, Tahira; McMaster University Faculty of Health Sciences, Clinical Epidemiology and Biostatistics<br>Roelofs, Marjan; University of Groningen, University Medical Center Groningen, department of General Practice and Elderly Care Medicine<br>Steffens, Martijn ; Isala Hospitals, urology<br>van der Worp, Henk; University of Groningen, University medical center groningen, Department General Practice and Elderly Care Medicine

Keywords:	Lower Urinary Tract Symptoms, Adrenergic alpha-Antagonists, Primary Health Care, Minimal Clinically Important Difference, Patient outcome assessment

SCHOLARONE™
Manuscripts

Minimal important differences in the International Prostate Symptom Score and Overactive Bladder Questionnaire in a primary care population

M.H. (Marco) Blanker[1], H.J. (Harma) Alma[1], T. (Tahira) Devji[2], M. (Marjan) Roelofs[1], M.G. (Martijn) Steffens[3], H. (Henk) van der Worp[1]

1. Department of General Practice and Elderly Care Medicine, University Medical Centre Groningen, University of Groningen
2. Department of Health Research Methods, Evidence and Impact, McMaster University, Canada
3. Department of Urology, Isala Clinics, Zwolle, The Netherlands

Corresponding author: Marco H. Blanker, MD PhD, general practitioner and epidemiologist, Department of General Practice and Elderly Care Medicine, University Medical Centre Groningen, University of Groningen, P.O. box 196, 9700 AD Groningen, The Netherlands, Phone: +31 6 14961542, Fax: +31 50 3632964

E-mail addresses: m.h.blanker@umcg.nl, h.j.alma@umcg.nl , devjits@mcmaster.ca , marjanroelofs@hotmail.com , m.steffens@isala.nl , h.van.der.worp@umcg.nl

Wordcount: 2,939

Keywords: Lower Urinary Tract Symptoms; Adrenergic alpha-Antagonists; Primary Health Care; Minimal Clinically Important Difference; Patient outcome assessment

For peer review only

ABSTRACT

Objectives: To determine the minimal important difference (MID) of the International Prostate Symptom Score (IPSS) and the Overactive Bladder Questionnaire short form (OAB-q SF) assessed in primary care among patients treated for LUTS.

Design: Single-arm, open-label observational cohort study with a 6-week follow-up.

Setting: Twenty-two pharmacies in the Netherlands.

Participants: We enrolled Dutch men with uncomplicated LUTS who received a new alpha-blocker prescription from their general practitioner or urologist.

Primary and secondary outcomes: The IPSS and OAB-q SF were completed before and after 6 weeks of therapy. At 6 weeks, men also completed the Perceived Global Impression of Improvement (PGI-I). The mean change scores of the IPSS and OAB-q SF were calculated for each PGI-I outcome category, with the category 'a little better' used to determine the MID. The standard error of measurement (SEM) was calculated for each questionnaire.

Results: In total, 165 men completed follow-up. The MID was 5.2 points (95%CI, 3.9–6.4; SEM 3.6) for the IPSS and 11.0 points (95%CI, 7.1–14.9; SEM 9.7) for the OAB-q SF. However, the MID for the IPSS was higher in men with severe baseline symptoms (7.1; 95%CI, 5.3–9.0) than in men with moderate baseline symptoms (3.2; 95%CI, 1.7–4.8).

Conclusion: In this study, the MID for the IPSS was considerably higher than the MID of 3.1 reported in the only other study on this topic, but may be due to methodological differences. Interpretation of the MID for the OAB-q SF is hampered by the overlap with the SEM. Future studies are needed to confirm our results because correlations between the PGI-I and symptom questionnaires were suboptimal.

ARTICLE SUMMARY

Strengths and limitations of this study:

- We assessed the minimal important difference (MID) of two frequently used questionnaires on lower urinary tract symptoms.

For peer review only

- Given that many men are treated in primary care, MID values for this setting are particularly important to inform evidence-based decision-making and to facilitate interpretation of the IPSS and OAB-q SF.
- Notably, the sample size of this study was small, which resulted in very low numbers of men being included in the PGI-I category 'very much better' or 'worsening of symptoms'.

Funding statement: This work was supported by The Hein Hogerzeil Foundation with an unrestricted grant. The Foundation was not involved in the design of this study, nor in the data collection, data analyses, interpretation of the outcomes, or the writing of this manuscript.

Conflict of interest statement: All authors have completed the ICMJE uniform disclosure form at www.icmje.org/coi_disclosure.pdf and declare: no support from any organisation for the submitted work; no financial relationships with any organisations that might have an interest in the submitted work in the previous three years; no other relationships or activities that could appear to have influenced the submitted work.

For peer review only

INTRODUCTION

Symptom severity is a key outcome for patients with LUTS and is most often evaluated by direct patient inquiry, using patient-reported outcome measures (PROMs). Although the IPSS is most often used for this purpose in both clinical trials and practice,[1-5] it fails to capture problematic symptoms such as urinary incontinence and urgency. Therefore, the Overactive Bladder Questionnaire (OAB-q) is increasingly being used to evaluate the treatment of overactive bladder,[6,7] with the short form (i.e., OAB-q SF) having the advantage of being less time consuming.[8] Together, both of the IPSS and OAB-q SF capture the spectrum of outcomes that are important to patients, but it is difficult to interpret the effects of an intervention expressed as mean scores or change scores over time.

The minimal important difference (MID) has proven invaluable when interpreting PROMs and could be of great value for both the IPSS and OAB-q SF.[9,10] To date, the MID has only been reported for the IPSS in a study conducted in secondary care among participants of clinical trials.[1,2] There has been no report of the MID for the OAB-q SF in any care setting. In countries like the Netherlands and the United Kingdom, most men with LUTS first visit their GP to seek treatment. Given that setting may affect the MID, possibly because of differences in baseline symptom severity,[9-11] we feel that it is important to assess the MID in a primary care setting. Knowledge about the MID in primary care will then provide invaluable data for interpreting treatment outcomes that may differ between primary and secondary care. In addition, evidence must be obtained from multiple studies to ensure that MID determinations are accurate.

In the current study, we aimed to determine the MIDs for both the IPSS and OAB-q SF in a patient cohort originating mainly from primary care.

METHODS

Study design

We conducted a prospective cohort study between January 2016 and April 2018. Baseline data for the IPSS and OAB-q SF were compared with follow-up data after 6 weeks of treatment. At

For peer review only

follow-up, participants also completed the Perceived Global Impression of Improvement (PGI-I) and we calculated the MID.

Participants

Adult men who visited a participating pharmacy in the north of the Netherlands were included if they received a new alpha-blocker prescription for uncomplicated LUTS from a GP or urologist. A prescription was defined as new if no alpha-blocker prescription had been given within the past year. The pharmacists checked if the alpha-blocker was indicated for LUTS and excluded men prescribed alpha-blockers for urinary tract stones or indwelling catheters. All participants provided written informed consent. The medical ethics committee of the University Medical Centre Groningen, the Netherlands, approved the study (number 2016.122).

Data collection

At baseline, before starting alpha-blocker therapy, all participants provided relevant descriptive data (e.g., age, duration of LUTS in months or years, and history of surgery for LUTS) and completed the Dutch versions of the IPSS and OAB-q SF. After 6 weeks, men who consented repeated the IPSS and OAB-q SF by postal invitation. At this time, we asked participants to complete the Perceived Global Impression of Improvement (PGI-I) questionnaire. [12]

Questionnaires

The IPSS contains seven questions and produces a total score that may range from 0 (no symptoms) to 35 points (maximum score). [1] IPSS scores are often categorized as no/mild symptoms (0–7 points), moderate symptoms (8–19 points), or severe symptoms (≥20 points). The MID for the IPSS is currently considered to be 3.1 points. [2] The OAB-q SF contains six questions on 6-point Likert-type scales, with the outcomes transformed to a 0–100 point scale in which higher scores indicate more severe symptoms. [8] The PGI-I is a validated generic tool for assessing overall improvement after treatment and is answered on a 7-point Likert-type scale, with the following options: 'very much better', 'much better', 'a little better', 'no change', 'a little worse', 'much worse' or 'very much worse'. We sent a reminder after two weeks to patients who did not respond to follow-up requests.

For peer review only

Data analyses

Baseline characteristics are reported as continuous variables and summarized as mean and sd or as median and interquartile range, depending on the distribution checked by the Shapiro–Wilk test. These characteristics were also compared between men with and without completed follow-up data to test for selective nonresponse. Next, the change scores of the IPSS and OAB-q SF were calculated by comparing the data between the baseline and follow-up questionnaires. Change scores were inverted to facilitate intuitive interpretation, with positive scores reflecting symptom improvement.

Various methods exist to determine the MID of questionnaires and are typically either anchor-based or distribution-based.[9, 13-15] The latter involve evaluating change in the PROM with the probability that the change occurred by chance, sample variation or measurement precision; however, they do not reflect patient perspectives.[14, 16] Thus, we used an anchor-based method,[9] in which we compared changes in the IPSS or OAB-q SF (PROM) with the PGI-I (the anchor). For each PGI-I category, we then present the mean change in scores from baseline to follow-up with the associated CIs. We defined the MID as the mean change in IPSS or OAB-q SF for the PGI-I category 'a little better', but still present the mean change scores for the other PGI-I categories.

The usefulness of anchor-based approaches depends on the relationship between the PROM and the anchor.[17-19] The anchor and PROM should be measuring the same or similar underlying constructs and should therefore be appreciably correlated. We therefore examined the Spearman correlation coefficients between the PGI-I and the IPSS and OAB-q SF for the baseline, follow-up and change data to ensure the anchor's validity. A correlation coefficient between the symptom change scores and the PGI-I of ≥0.50, and an equal and opposite correlation of the PGI-I with the baseline score and the follow-up score, were considered ideal and likely to yield trustworthy MID estimates.[17-19]

To test the impact of baseline symptom severity on the distribution of results, a stratified analysis was performed for the IPSS categories 'moderate symptoms' and 'severe

For peer review only - http://bmjopen.bmj.com/site/about/guidelines.xhtml

symptoms' because previous research has shown that such stratification has a large impact.[2] No such categories have been defined for the OAB-q SF, so we did not perform a similar analysis for this questionnaire. Subgroup analyses were also performed with participants who received their prescription from their GP, allowing us to provide data that focused on the primary care setting. Finally, we checked if the MID exceeded the measurement error.[15, 17] For this, we calculated the standard error of measurement (SEM) as follows: [sd × (1 – reliability)$^{1/2}$]. Cronbach's alpha was used as the reliability measure.[20]

The complete data set was used without imputing missing data. All analyses were performed using IBM SPSS version 25.0 (IBM Corp., Armonk, NY, USA), and we considered a p-value <0.05 to be statistically significant.

Patient and public involvement

This study was performed without patient involvement. We did not invite patients to comment on the study design nor did we consult them to interpret the results. Patients were not invited to contribute to the writing or editing of this document for readability or accuracy.

RESULTS

A total of 251 men completed the baseline questionnaires, of which 165 also completed the follow-up questionnaires. The baseline characteristics of men with and without follow-up data are shown in Table 1, with no statistically significant differences found between these groups. Notably, 86.3% of men received their prescription from a GP and the remainder received it from a urologist.

There were mean improvements in the IPSS and OAB-q SF scores during the study of 5.8 (sd 6.7) and 11.8 points (sd 17.4), respectively. Between baseline and follow-up at 6 weeks, the mean IPSS score changed from 19.1 (sd 6.8) to 13.3 (sd 6.5) and the mean OAB-q SF score changed from 39.7 (sd 19.2) to 27.9 (sd 16.9). The PGI-I outcomes are shown in Table 2 and indicate that most men reported that they were 'a little better' or 'much better' (74.7%), whilst only 23.5% perceived no change. Only three men (1.8%) reported 'worsened' symptoms, and

For peer review only - http://bmjopen.bmj.com/site/about/guidelines.xhtml

none of the participants reported 'much worsened' or 'very much worsened' symptoms.

Table 2 also shows the distribution of changes in the IPSS and OAB-q SF for each PGI-I category. The MID for the IPSS was 5.2 points (95% CI, 3.9–6.4) and the PGI-I outcomes 'no change' and 'much better' corresponded to IPSS symptom changes of 3.1 points (95% CI, 1.1–5.1) and 8.7 points (95% CI, 6.8–10.7), respectively. The MID for the OAB-q SF was 11.0 points (95% CI, 7.1 to 14.9) and the PGI-I outcomes 'no change' and 'much better' corresponded with mean improvements of 3.0 points (95% CI, -2.3 to 8.4) and 19.1 points (95% CI, 14.3–24.0), respectively. The Spearman correlation coefficients were then calculated between the PGI-I and both the IPSS and the OAB-q SF. The correlation was -0.51 for the PGI-I and baseline IPSS, 0.43 for the PGI-I and follow-up IPSS and 0.38 for the PGI-I and change in IPSS. The corresponding correlations for the OAB-q SF were -0.09 at baseline, 0.36 at follow-up and 0.42 for the change.

Subgroup analyses of data for men with a prescription from a GP found no relevant differences, with MID values of 5.4 for the IPSS and 11.2 for the OAB-q SF (Table 3). Stratified analysis of baseline data revealed that men with severe symptoms had higher MID values for the IPSS, reaching 7.1 (95% CI, 5.3–9.0), compared with the MID value of 3.2 (95% CI, 1.7–4.8) for men with moderate symptoms (Table 4).

Finally, the SEM was 3.6 for the IPSS and 9.7 for the OAB-q SF.

DISCUSSION

We estimated the MID for two questionnaires that are often used to assess male LUTS in primary care. However, whereas the SEM of the IPSS was less than the 95% CI of the MID (5.2 points; 95% CI, 3.9–6.4; SEM 3.6), the SEM of the OAB-q SF fell within the 95% CI of the MID (11 points; 95% CI, 7.1–14.9; SEM 9.7). Thus, we can only conclude that the outcomes for the IPSS were unlikely to have occurred because of chance or measurement imprecision. Given that many questionnaires have used multiple MID values, we were surprised to find only one previous estimate of the MID for the IPSS in the literature.[1, 2] Our study therefore adds relevant information in the primary care setting for clinicians and guideline developers.

For peer review only - http://bmjopen.bmj.com/site/about/guidelines.xhtml

Our results for the IPSS were different to those of the seminal study on this topic performed by Barry et al. in secondary care.[1, 2] In that study, the MID of 3.1 points (sd 0.27) fell within the 95% CI of the 'no change' group, but outside the CI of the 'a little better' group, suggesting a likely underestimation of the real value. In the current study, there was also some overlap between the CIs of the 'no change' and the 'a little better' group, though this was within a change of only 3.9 to 5.1 points. Given that treatment is typically in primary care, we have therefore provided additional data that is applicable to most men with LUTS. Nevertheless, the differences in outcomes compared with the study by Barry et al. need to be explained. It is our contention that three methodological differences account for these differences.

First, Barry et al. compared patients between baseline and follow-up after 13 weeks. By contrast, the follow-up period in the current study was only 6 weeks. Although this difference of 7 weeks may have affected the ability of patients to recall their prior health state accurately, the true impact of this remains unclear. Change scores may also have been influenced by the natural variation that occurs in symptom severity over time.

Second, in the research by Barry et al., a global assessment of patient improvement was used at baseline. This included a 5-point scale with the options 'marked improvement', 'moderate improvement', 'slight improvement', 'no improvement', and 'worse' for which the exact question was not reported. In our study, we used a 7-point Likert-type scale that ranged from 'very much better' to 'very much worsened'. We considered that this difference probably had no more than a marginal impact given that the positive outcome categories were comparable in both studies. Notably, none of the participants in our study reported that the symptoms had 'very' or 'very much' worsened.

Third, we mainly included men from primary care, rather than men solely from secondary care. Although it is generally thought that men in primary care have fewer symptoms, our men tended to have more severe symptoms (IPSS score >19) than in the study by Barry et al. (45% versus 25%). Barry et al. also reported that baseline severity had a major impact on the MID, but when we compare their stratified analysis with ours, we had higher mean change

For peer review only - http://bmjopen.bmj.com/site/about/guidelines.xhtml

scores for each PGI-I category. This might be explained by the fact that we only included men who actually used an alpha-blocker. In contrast to this focused approach, Barry et al. used data for all participants in a large, randomized, double-blind trial of four treatment strategies for male LUTS. In their study, a lower MID could therefore have resulted from the inclusion of patients receiving placebo, finasteride, terazosin or combination therapy given that the efficacy of alpha-blockers exceeds that of both placebo and finasteride. The use of blinding meant that men who used placebo or finasteride may have overestimated their subjective improvement, whilst alpha-blocker users may have underestimated their subjective improvement. Given that the IPSS objectively counts symptoms, the placebo and finasteride users would experience a smaller change in the IPSS whereas the active drug users would experience a larger effect. Although the actual impact of each intervention is unknown, researchers in other fields have made similar observations. [10, 11, 21]

We were unable to find any prior estimates of the MID for the OAB-q SF in the literature. Our finding that 11.0 points (95% CI, 7.1–14.9) indicates a clinically relevant change is therefore a novel and important finding, but one for which the reliability will need to be assessed in other studies. We recognize that alpha-blockers are not specifically indicated for the treatment of overactive bladder, but we contend that there is a considerable overlap with LUTS unrelated to overactive bladder. Indeed, guidelines suggest prescribing alpha-blockers for most men with LUTS who request active treatment. This is because these agents have a rapid onset of action, good efficacy, and low rate and severity of adverse events. [3-5] We recommend further study to determine the MID in men with specific symptoms of overactive bladder treated with anticholinergics or beta-3 agonists.

Some limitations need to be considered when assessing our results. Notably, the sample size of this study was small, which resulted in very low numbers of men being included in the PGI-I category ‘very much better’. For that category, the mean change scores for both questionnaires showed very wide CIs. The same holds for the categories linked to symptom worsening. In those categories, a trial discontinuation may be more suitable for reliable

For peer review only - http://bmjopen.bmj.com/site/about/guidelines.xhtml

estimates. The sample size might also explain why the CI of the MID estimate for the OAB-q SF included the SEM.

Another limitation is reflected by difficulties we encountered with some of the associations between the PGI-I and the two PROM questionnaires. For the IPSS, the follow-up IPSS and PGI-I scores correlated better than with IPSS change and PGI-I scores, suggesting that this rating only reflected the current status, which in turn, decreases confidence in the MID estimate. For the OAB-q SF, the correlation coefficient between the baseline OAB-q SF and PGI-I scores was opposite in magnitude to that for the follow-up OAB-q SF and PGI-I scores. With both questionnaires, the correlation coefficients for the change scores were lower than the threshold of 0.5 that we set a priori. [18] High correlation coefficients are preferred between the anchor and the change in PROM, though some researchers have suggested applying lower thresholds. [22] Still, even the high correlation coefficients are insufficient to confirm that the transition rating is in fact measuring change as opposed to current health status. [18] Unfortunately, Barry et al. did not report the correlation coefficients between the IPSS and the anchor, [2] which is consistent with most other research for PROMs. [23] Given the suboptimal relationship between the PROM and the anchor, we must stress that the estimates obtained for the MID should be interpreted with caution and should be confirmed in future investigations with larger samples.

In conclusion, this study is the first to define MID values for two important PROMs used to evaluate the effectiveness of treatment for male LUTS in primary care. Given that many men are treated in primary care, MID values for this setting are particularly important to inform evidence-based decision-making and to facilitate interpretation of the IPSS and OAB-q SF. Moreover, we consider that this study emphasizes the importance of the MID to individual patients in daily practice. We defined the MID based on the PGI-I outcome 'a little better' in the present study, but patients may expect 'much better' as an outcome when starting therapy. To date, most outcomes of alpha-blocker and other drug treatments for male LUTS have been expressed as the mean IPSS change scores. In the vast majority of studies [3], difference in IPSS changes between active treatment and placebo have approached, but not exceeded, the

For peer review only - http://bmjopen.bmj.com/site/about/guidelines.xhtml

previously reported MID of 3.1.[1,2] Applying a threshold for improvement of 5.2 points, as described in our study, may change the interpretation of those studies.

Acknowledgements

We are grateful for the assistance of Tom Vermist in collecting the data. The authors thank all patients and collaborating pharmacies for their participation, as well as Dr Robert Sykes (www.doctored.org.uk) for providing editorial services.

Author contributions

The corresponding author attests that all listed authors meet authorship criteria and that no others meeting the criteria have been omitted.

MHB and MGS initiated the study. Acquisition of the data was done by MHB and MR. Analysis and interpretation of the data was done by MHB, HJA, TD and HW. MHB wrote the manuscript with extensive support from HJA and TD.

All authors critically reviewed the manuscript.

Data sharing statement

Data collected for this study will be available from the corresponding author upon request.

REFERENCES

1 Barry MJ, Fowler FJ,Jr, O'Leary MP, et al. The American Urological Association symptom index for benign prostatic hyperplasia. The Measurement Committee of the American Urological Association*. J Urol* 1992;148:1549,57; discussion 1564.

2 Barry MJ, Williford WO, Chang Y, et al. Benign prostatic hyperplasia specific health status measures in clinical research: how much change in the American Urological Association symptom index and the benign prostatic hyperplasia impact index is perceptible to patients?*. J Urol* 1995;154:1770-4.

3 National Clinical Guideline Centre (NICE). Lower urinary tract symptoms in men: management (Clinical guideline CG97) 2015;CG97 doi:http://www.nice.org.uk/nicemedia/live/12984/48554/48554.pdf;.

For peer review only - http://bmjopen.bmj.com/site/about/guidelines.xhtml

4 Gratzke C, Bachmann A, Descazeaud A, et al. EAU Guidelines on the Assessment of Non-neurogenic Male Lower Urinary Tract Symptoms including Benign Prostatic Obstruction. *Eur Urol* 2015;67:1099-109 doi:10.1016/j.eururo.2014.12.038 [doi].

5 Blanker MH, Klomp MA, van den Donk M, et al. Summary of the NHG practice guideline 'Lower urinary tract symptoms in men'. *Ned Tijdschr Geneeskd* 2013;157:A6178.

6 Coyne K, Revicki D, Hunt T, et al. Psychometric validation of an overactive bladder symptom and health-related quality of life questionnaire: the OAB-q. *Qual Life Res* 2002;11:563-74.

7 Coyne KS, Matza LS, Thompson CL. The responsiveness of the Overactive Bladder Questionnaire (OAB-q). *Qual Life Res* 2005;14:849-55.

8 Coyne KS, Thompson CL, Lai JS, et al. An overactive bladder symptom and health-related quality of life short-form: validation of the OAB-q SF. *Neurourol Urodyn* 2015;34:255-63 doi:10.1002/nau.22559 [doi].

9 Jaeschke R, Singer J, Guyatt GH. Measurement of health status. Ascertaining the minimal clinically important difference. *Control Clin Trials* 1989;10:407-15.

10 Terwee CB, Roorda LD, Dekker J, et al. Mind the MIC: large variation among populations and methods. *J Clin Epidemiol* 2010;63:524-34 doi:10.1016/j.jclinepi.2009.08.010 [doi].

11 Olsen MF, Bjerre E, Hansen MD, et al. Minimum clinically important differences in chronic pain vary considerably by baseline pain and methodological factors: systematic review of empirical studies. *J Clin Epidemiol* 2018;101:87,106.e2 doi:S0895-4356(18)30124-0 [pii].

12 Viktrup L, Hayes RP, Wang P, et al. Construct validation of patient global impression of severity (PGI-S) and improvement (PGI-I) questionnaires in the treatment of men with lower urinary tract symptoms secondary to benign prostatic hyperplasia. *BMC Urol* 2012;12:30,2490-12-30 doi:10.1186/1471-2490-12-30 [doi].

13 Angst F, Aeschlimann A, Angst J. The minimal clinically important difference raised the significance of outcome effects above the statistical level, with methodological implications for future studies. *J Clin Epidemiol* 2017;82:128-36 doi:S0895-4356(16)30776-4 [pii].

14 Crosby RD, Kolotkin RL, Williams GR. Defining clinically meaningful change in health-related quality of life. *J Clin Epidemiol* 2003;56:395-407 doi:S0895435603000441 [pii].

For peer review only

15 Norman GR, Sloan JA, Wyrwich KW. Interpretation of changes in health-related quality of life: the remarkable universality of half a standard deviation. *Med Care* 2003;41:582-92 doi:10.1097/01.MLR.0000062554.74615.4C [doi].

16 de Vet HC, Terwee CB. The minimal detectable change should not replace the minimal important difference. *J Clin Epidemiol* 2010;63:804,5; author reply 806 doi:10.1016/j.jclinepi.2009.12.015 [doi].

17 Cella D, Hahn EA, Dineen K. Meaningful change in cancer-specific quality of life scores: differences between improvement and worsening. *Qual Life Res* 2002;11:207-21.

18 Guyatt GH, Norman GR, Juniper EF, et al. A critical look at transition ratings. *J Clin Epidemiol* 2002;55:900-8 doi:S0895435602004353 [pii].

19 Guyatt GH, Jaeschke RJ. Reassessing quality-of-life instruments in the evaluation of new drugs. *Pharmacoeconomics* 1997;12:621-6 doi:10.2165/00019053-199712060-00002 [doi].

20 Wyrwich KW, Tierney WM, Wolinsky FD. Using the standard error of measurement to identify important changes on the Asthma Quality of Life Questionnaire. *Qual Life Res* 2002;11:1-7.

21 Devji T, Guyatt GH, Lytvyn L, et al. Application of minimal important differences in degenerative knee disease outcomes: a systematic review and case study to inform BMJ Rapid Recommendations. *BMJ Open* 2017;7:e015587,2016-015587 doi:10.1136/bmjopen-2016-015587 [doi].

22 Revicki D, Hays RD, Cella D, et al. Recommended methods for determining responsiveness and minimally important differences for patient-reported outcomes. *J Clin Epidemiol* 2008;61:102-9 doi:10.1016/j.jclinepi.2007.03.012 [doi].

23 Alma H, de Jong C, Tsiligianni I, et al. Clinically relevant differences in COPD health status: systematic review and triangulation. *Eur Respir J* 2018;52:1800412 doi:10.1183/13993003.00412.

For peer review only

Table 1. Baseline characteristics of all participants and participants who dropped out after the baseline questionnaire

	Full participants (n = 165)	Drop out after baseline (n = 86)	p-value
Age (mean ± sd)	66.7±9.7	65.4±12.1	0.42*
Prescription from GP (%)	86.3	83.2	0.55#
IPSS score (mean ± sd)	19.1±6.8	17.6±6.5	0.11*
IPSS categories (%)			0.12#
- none/mild	3.7	6.4	
- moderate	50.9	61.5	
- severe	45.3	32.1	
IPSS Quality of Life (median | IQR)	4.0 | 2.0	4.0 | 2.0	0.52§
OAB-q SF (mean ± sd)	39.8±19.2	40.7±18.1	0.70*
Duration of LUTS in months (median | IQR)	24.0 | 37	12.0 | 33	0.11§
History of surgery for LUTS (%)	1.2	3.8	0.19#

IQR: interquartile range. P-values refer to: * student-t-test; # chi-squared test; § Mann–Whitney U test.

Abbreviations: OAB-q SF, Overactive Bladder Questionnaire short form.

For peer review only

For peer review only - http://bmjopen.bmj.com/site/about/guidelines.xhtml

Table 2. Change scores for the IPSS and OAB-q SF by PGI-I outcomes

**PGI-I outcome**	**N (%)**	**IPSS**	Missing	**OAB-q SF**	Missing
Very much better	6 (3.6)	13.4 (2.9;23.9)	1	23.8 (2.3;45.3)	0
Much better	50 (30.3)	8.7 (6.8;10.7)	2	19.1 (14.3;24.0)	3
**A little better**	**68 (41.2)**	**5.2 (3.9;6.4)**	**3**	**11.0 (7.1;14.9)**	**4**
No change	38 (23.0)	3.1 (1.1;5.1)	0	3.0 (-2.3;8.4)	4
A little worse	3 (1.8)	-5.0 (-30.9;20.9)	0	-9.7 (-81.7;62.4)	0

Change in IPSS and OAB-q SF scores were estimated by comparing symptom scores between baseline and 6 weeks. Mean change and 95% CIs are presented. Outcomes are inverted so that positive changes reflect symptom improvement. The PGI-I category 'a little better' reflects the MID for both questionnaires. None of the participants scored 'much worsened' or 'very much worsened' on the PGI-I. Abbreviations: MID, Minimal important difference; OAB-q SF, Overactive Bladder Questionnaire short form; PGI-I, Perceived Global Impression of Improvement.

For peer review only

Table 3. Change scores for the IPSS and OAB-q SF by PGI-I outcomes: subgroup analysis for GP prescriptions

PGI-I outcome	N (%)	IPSS	Missing	OAB-q SF	Missing
Very much better	4 (3.1)	18.0 (1.7;34.3)	1	30.8 (-0.9;62.4)	0
Much better	39 (30.2)	9.2 (7.0;11.5)	2	19.9 (14.8;24.9)	2
**A little better**	**57 (44.2)**	**5.4 (4.0;6.7)**	**3**	**11.2 (7.0;15.4)**	**3**
No change	27 (20.9)	3.1 (0.5;5.6)	0	3.3 (-3.4;9.9)	3
A little worse	2 (1.6)	-8.5 (-11.6.5; 99.5)	0	-16.5 (-353.2;320.2)	0

Change in IPSS and OAB-q SF scores were estimated by comparing symptom scores between baseline and 6 weeks. Mean change and 95% CIs are presented. Outcomes are inverted so that positive changes reflect symptom improvement. The PGI-I category 'a little better' reflects the MID for both questionnaires. None of the participants scored 'much worsened' or 'very much worsened' on the PGI-I. Abbreviations: MID, Minimal important difference; OAB-q SF, Overactive Bladder Questionnaire short form; PGI-I, Perceived Global Impression of Improvement.

For peer review only

Table 4. Change scores for the IPSS by PGI-I outcomes

	Moderate symptoms (n = 88)			Severe symptoms (n = 73)	
**PGI-I outcome**	**N (%)**	**Change**	**Missing**	**N (%)**	**Change**
Very much better	3 (3.4)	6.5 (-50.7;63.7)	1	3 (4.1)	18 (1.7;34.3)
Much better	30 (34.1)	5.6 (3.7;7.5)	1	19 (26.0)	13.5 (10.5;16.5)
**A little better**	33 (37.5)	3.2 (1.7;4.8)	0	32 (43.8)	7.1 (5.3;9.0)
No change	19 (21.6)	1.3 (-1.7;4.3)	0	19 (26.0)	4.9 (2.3;7.6)
A little worse	3 (3.4)	-5.0 (-31.0;20.9)	0	0 (0.0)	–

These results are stratified by baseline symptom severity on the IPSS: moderate symptoms are scores of 8–19 and severe symptoms are scores of ≥20. Change in IPSS scores were estimated by comparing symptom scores between baseline and 6 weeks. Mean change and 95% CIs are presented. Outcomes are inverted so that positive changes reflect symptom improvement. The PGI-I category 'a little better' reflects the MID for both questionnaires. None of the participants scored 'much worsened' or 'very much worsened' on the PGI-I. Abbreviations: MID, Minimal important difference; OAB-q SF, Overactive Bladder Questionnaire short form; PGI-I, Perceived Global Impression of Improvement.

For peer review only

For peer review only

For peer review only - http://bmjopen.bmj.com/site/about/guidelines.xhtml

STROBE Statement—Checklist of items that should be included in reports of ***cohort studies***

	Item No	Recommendation	Page No
**Title and abstract**	1	(*a*) Indicate the study's design with a commonly used term in the title or the abstract	2
		(*b*) Provide in the abstract an informative and balanced summary of what was done and what was found	2
**Introduction**			
Background/rationale	2	Explain the scientific background and rationale for the investigation being reported	4
Objectives	3	State specific objectives, including any prespecified hypotheses	4
**Methods**			
Study design	4	Present key elements of study design early in the paper	4-5
Setting	5	Describe the setting, locations, and relevant dates, including periods of recruitment, exposure, follow-up, and data collection	5
Participants	6	(*a*) Give the eligibility criteria, and the sources and methods of selection of participants. Describe methods of follow-up	5
		(*b*) For matched studies, give matching criteria and number of exposed and unexposed	
Variables	7	Clearly define all outcomes, exposures, predictors, potential confounders, and effect modifiers. Give diagnostic criteria, if applicable	5-6
Data sources/ measurement	8*	For each variable of interest, give sources of data and details of methods of assessment (measurement). Describe comparability of assessment methods if there is more than one group	5-6
Bias	9	Describe any efforts to address potential sources of bias	7
Study size	10	Explain how the study size was arrived at	-
Quantitative variables	11	Explain how quantitative variables were handled in the analyses. If applicable, describe which groupings were chosen and why	6
Statistical methods	12	(*a*) Describe all statistical methods, including those used to control for confounding	6-7
		(*b*) Describe any methods used to examine subgroups and interactions	6
		(*c*) Explain how missing data were addressed	7
		(*d*) If applicable, explain how loss to follow-up was addressed	
		(*e*) Describe any sensitivity analyses	
**Results**			
Participants	13*	(a) Report numbers of individuals at each stage of study—eg numbers potentially eligible, examined for eligibility, confirmed eligible, included in the study, completing follow-up, and analysed	7
		(b) Give reasons for non-participation at each stage	
		(c) Consider use of a flow diagram	
Descriptive data	14*	(a) Give characteristics of study participants (eg demographic, clinical, social) and information on exposures and potential confounders	7
		(b) Indicate number of participants with missing data for each variable of interest	7
		(c) Summarise follow-up time (eg, average and total amount)	7
Outcome data	15*	Report numbers of outcome events or summary measures over time	-

For peer review only - http://bmjopen.bmj.com/site/about/guidelines.xhtml

Main results	16	(*a*) Give unadjusted estimates and, if applicable, confounder-adjusted estimates and their precision (eg, 95% confidence interval). Make clear which confounders were adjusted for and why they were included	7-8
		(*b*) Report category boundaries when continuous variables were categorized	7-8
		(*c*) If relevant, consider translating estimates of relative risk into absolute risk for a meaningful time period	
Other analyses	17	Report other analyses done—eg analyses of subgroups and interactions, and sensitivity analyses	7-8
**Discussion**			
Key results	18	Summarise key results with reference to study objectives	8
Limitations	19	Discuss limitations of the study, taking into account sources of potential bias or imprecision. Discuss both direction and magnitude of any potential bias	10-11
Interpretation	20	Give a cautious overall interpretation of results considering objectives, limitations, multiplicity of analyses, results from similar studies, and other relevant evidence	8-11
Generalisability	21	Discuss the generalisability (external validity) of the study results	11
**Other information**			
Funding	22	Give the source of funding and the role of the funders for the present study and, if applicable, for the original study on which the present article is based	3

*Give information separately for exposed and unexposed groups.

Note: An Explanation and Elaboration article discusses each checklist item and gives methodological background and published examples of transparent reporting. The STROBE checklist is best used in conjunction with this article (freely available on the Web sites of PLoS Medicine at http://www.plosmedicine.org/, Annals of Internal Medicine at http://www.annals.org/, and Epidemiology at http://www.epidem.com/). Information on the STROBE Initiative is available at http://www.strobe-statement.org.

For peer review only

BMJ Open

Determining the minimal important differences in the International Prostate Symptom Score and Overactive Bladder Questionnaire: results from an observational cohort study in primary care

Journal:	*BMJ Open*
Manuscript ID	bmjopen-2019-032795.R1
Article Type:	Original research
Date Submitted by the Author:	14-Oct-2019
Complete List of Authors:	Blanker, Marco; University of Groningen, University Medical Center Groningen, department of General Practice and Elderly Care Medicine<br>Alma, Harma; University of Groningen, University Medical Center Groningen, Department of General Practice and Elderly Care Medicine; University of Groningen, University Medical Center Groningen, Groningen Research Institute for Asthma and COPD (GRIAC)<br>Devji, Tahira; McMaster University Faculty of Health Sciences, Clinical Epidemiology and Biostatistics<br>Roelofs, Marjan; University of Groningen, University Medical Center Groningen, department of General Practice and Elderly Care Medicine<br>Steffens, Martijn ; Isala Hospitals, urology<br>van der Worp, Henk; University of Groningen, University medical center groningen, Department General Practice and Elderly Care Medicine
**Primary Subject Heading**:	General practice / Family practice
Secondary Subject Heading:	Urology, Epidemiology
Keywords:	Lower Urinary Tract Symptoms, Adrenergic alpha-Antagonists, Primary Health Care, Minimal Clinically Important Difference, Patient outcome assessment

SCHOLARONE™
Manuscripts

Determining the minimal important differences in the International Prostate Symptom Score and Overactive Bladder Questionnaire: results from an observational cohort study in primary care

M.H. (Marco) Blanker[1], H.J. (Harma) Alma[1], T. (Tahira) Devji[2], M. (Marjan) Roelofs[1], M.G. (Martijn) Steffens[3], H. (Henk) van der Worp[1]

1. Department of General Practice and Elderly Care Medicine, University Medical Centre Groningen, University of Groningen
2. Department of Health Research Methods, Evidence and Impact, McMaster University, Canada
3. Department of Urology, Isala Clinics, Zwolle, The Netherlands

Corresponding author: Marco H. Blanker, MD PhD, general practitioner and epidemiologist, Department of General Practice and Elderly Care Medicine, University Medical Centre Groningen, University of Groningen, P.O. box 196, 9700 AD Groningen, The Netherlands, Phone: +31 6 14961542, Fax: +31 50 3632964

E-mail addresses: m.h.blanker@umcg.nl, h.j.alma@umcg.nl , devjits@mcmaster.ca , marjanroelofs@hotmail.com , m.steffens@isala.nl , h.van.der.worp@umcg.nl

Wordcount: 3,116

Keywords: Lower Urinary Tract Symptoms; Adrenergic alpha-Antagonists; Primary Health Care; Minimal Clinically Important Difference; Patient outcome assessment

For peer review only

ABSTRACT

Objectives: To determine the minimal important difference (MID) of the International Prostate Symptom Score (IPSS) and the Overactive Bladder Questionnaire short form (OAB-q SF) assessed in primary care among patients treated for lower urinary tract symptoms (LUTS).

Design: Single-arm, open-label observational cohort study with a 6-week follow-up.

Setting: Twenty-two pharmacies in the Netherlands.

Participants: We enrolled Dutch men with uncomplicated LUTS who received a new alpha-blocker prescription from their general practitioner or urologist.

Primary and secondary outcomes: The IPSS and OAB-q SF were completed before and after 6 weeks of therapy. At 6 weeks, men also completed the Patient Global Impression of Improvement (PGI-I). The mean change scores of the IPSS and OAB-q SF were calculated for each PGI-I outcome category, with the category 'a little better' used to determine the MID. The standard error of measurement (SEM) was calculated for each questionnaire.

Results: In total, 165 men completed follow-up. The MID was 5.2 points (95%CI, 3.9–6.4; SEM 3.6) for the IPSS and 11.0 points (95%CI, 7.1–14.9; SEM 9.7) for the OAB-q SF. For both questionnaires, confidence intervals showed an overlap with the no-change categories. However, the MID for the IPSS was higher in men with severe baseline symptoms (7.1; 95%CI, 5.3–9.0) than in men with moderate baseline symptoms (3.2; 95%CI, 1.7–4.8).

Conclusion: In this study, the MID for the IPSS was considerably higher than the MID of 3.1 reported in the only other study on this topic, but may be due to methodological differences. Interpretation of the MID for the OAB-q SF is hampered by the overlap with the SEM. Future studies are needed to confirm our results because correlations between the PGI-I and symptom questionnaires were suboptimal.

ARTICLE SUMMARY

Strengths and limitations of this study:

- We assessed the minimal important difference (MID) of two frequently used questionnaires

For peer review only

on lower urinary tract symptoms.

- Given that many men are treated in primary care, MID values for this setting are particularly important to inform evidence-based decision-making and to facilitate interpretation of the IPSS and OAB-q SF.
- Notably, the sample size of this study was small, which resulted in very low numbers of men being included in the PGI-I category 'very much better' or 'worsening of symptoms', and may clarify the small overlap of the confidence intervals with the no-change category.

Funding statement: This work was supported by The Hein Hogerzeil Foundation with an unrestricted grant. The Foundation was not involved in the design of this study, nor in the data collection, data analyses, interpretation of the outcomes, or the writing of this manuscript.

Conflict of interest statement: All authors have completed the ICMJE uniform disclosure form at www.icmje.org/coi_disclosure.pdf and declare: no support from any organisation for the submitted work; no financial relationships with any organisations that might have an interest in the submitted work in the previous three years; no other relationships or activities that could appear to have influenced the submitted work.

For peer review only

INTRODUCTION

Symptom severity is a key outcome for patients with LUTS and is most often evaluated by direct patient inquiry, using patient-reported outcome measures (PROMs). Although the IPSS is most often used for this purpose in both clinical trials and practice,[1-5] it fails to capture problematic symptoms such as urinary incontinence and urgency. Therefore, the Overactive Bladder Questionnaire (OAB-q) is increasingly being used to evaluate the treatment of overactive bladder,[6,7] with the short form (i.e., OAB-q SF) having the advantage of being less time consuming.[8] Together, both of the IPSS and OAB-q SF capture the spectrum of outcomes that are important to patients, but it is difficult to interpret the effects of an intervention expressed as mean scores or change scores over time.

The minimal important difference (MID) has proven invaluable when interpreting PROMs and could be of great value for both the IPSS and OAB-q SF.[9,10] To date, the MID has only been reported for the IPSS in a study conducted in secondary care among participants of clinical trials.[1,2] There has been no report of the MID for the OAB-q SF in any care setting. In countries like the Netherlands and the United Kingdom, most men with LUTS first visit their GP to seek treatment. Given that setting may affect the MID, possibly because of differences in baseline symptom severity,[9-11] we feel that it is important to assess the MID in a primary care setting. Knowledge about the MID in primary care will then provide invaluable data for interpreting treatment outcomes that may differ between primary and secondary care. In addition, evidence must be obtained from multiple studies to ensure that MID determinations are accurate.

In the current study, we aimed to determine the MIDs for both the IPSS and OAB-q SF in a patient cohort originating mainly from primary care.

METHODS

Study design

We conducted a prospective cohort study between January 2016 and April 2018.[12] Baseline data for the IPSS and OAB-q SF were compared with follow-up data after 6 weeks of treatment.

For peer review only - http://bmjopen.bmj.com/site/about/guidelines.xhtml

At follow-up, participants also completed the Patient Global Impression of Improvement (PGI-I) and we calculated the MID.

Participants

Adult men who visited a participating pharmacy in the north of the Netherlands were included if they received a new alpha-blocker prescription for uncomplicated LUTS from a GP or urologist. A prescription was defined as new if no alpha-blocker prescription had been given within the past year. The pharmacists checked if the alpha-blocker was indicated for LUTS and excluded men prescribed alpha-blockers for urinary tract stones or indwelling catheters. All participants provided written informed consent. The medical ethics committee of the University Medical Centre Groningen, the Netherlands, approved the study (number 2016.122).

Data collection

At baseline, before starting alpha-blocker therapy, all participants provided relevant descriptive data (e.g., age, duration of LUTS in months or years, and history of surgery for LUTS) and completed the Dutch versions of the IPSS and OAB-q SF. After 6 weeks, men who consented repeated the IPSS and OAB-q SF by postal invitation. At this time, we asked participants to complete the Patient Global Impression of Improvement (PGI-I) questionnaire.[13] The period of six weeks was chosen as clinical effects of alpha-blockers take a few weeks to develop fully, but significant efficacy over placebo can occur within hours to days.[4, 14]

Questionnaires

The IPSS contains seven questions and produces a total score that may range from 0 (no symptoms) to 35 points (maximum score).[1] Each question has response options ranging from 0 to five, with higher scores reflecting more severe symptoms. IPSS scores are often categorized as no/mild symptoms (0–7 points), moderate symptoms (8–19 points), or severe symptoms (≥20 points). The IPSS was internally consistent (Cronbach's alpha = 0.86) and has excellent test-retest reliability (r = 0,92).[1] The MID for the IPSS is currently considered to be 3.1 points.[2] The OAB-q SF contains six questions on 6-point Likert-type scales, with the outcomes transformed to a 0–100 point scale in which higher scores indicate more severe symptoms.[8]

For peer review only

This scale demonstrated good convergent validity, discriminant validity, internal reliability, reproducibility, and responsiveness to change.[8] The PGI-I is a validated generic tool for assessing overall improvement after treatment and is answered on a 7-point Likert-type scale, with the following options: 'very much better', 'much better', 'a little better', 'no change', 'a little worse', 'much worse' or 'very much worse'.[13,15] Full versions of these questionnaires are presented as supplementary file 1.

We sent a reminder after two weeks to patients who did not respond to follow-up requests.

Data analyses

Baseline characteristics are reported as continuous variables and summarized as mean and SD or as median and interquartile range, depending on the distribution checked by the Shapiro–Wilk test. These characteristics were also compared between men with and without completed follow-up data to test for selective nonresponse. Next, the change scores of the IPSS and OAB-q SF were calculated by comparing the data between the baseline and follow-up questionnaires. Change scores were inverted to facilitate intuitive interpretation, with positive scores reflecting symptom improvement.

Various methods exist to determine the MID of questionnaires and are typically either anchor-based or distribution-based.[9,16-18] The latter involve evaluating change in the PROM with the probability that the change occurred by chance, sample variation or measurement precision; however, they do not reflect patient perspectives.[17,19] Thus, we used an anchor-based method,[9] in which we compared changes in the IPSS or OAB-q SF (PROM) with the PGI-I (the anchor). For each PGI-I category, we then present the mean change in scores from baseline to follow-up with the associated CIs. We defined the MID as the mean change in IPSS or OAB-q SF for the PGI-I category 'a little better', but still present the mean change scores for the other PGI-I categories.

The usefulness of anchor-based approaches depends on the relationship between the PROM and the anchor.[20-22] The anchor and PROM should be measuring the same or similar underlying constructs and should therefore be appreciably correlated. Correlations between

For peer review only - http://bmjopen.bmj.com/site/about/guidelines.xhtml

questionnaire change scores and the anchor PGI-I should be obviously strong, as else these measure different concepts. Correlations between the anchor PGI-I and the baseline and follow-up questionnaire scores are performed to check for a possible response shift. Mostly anchor PGI-I scores seem correlated with follow-up scores (due to response shift). We therefore examined the Spearman correlation coefficients between the PGI-I and the IPSS and OAB-q SF for the baseline, follow-up and change data to ensure the anchor's validity. A correlation coefficient between the symptom change scores and the PGI-I of ≥0.50, and an equal and opposite correlation of the PGI-I with the baseline score and the follow-up score, were considered ideal and likely to yield trustworthy MID estimates.[20-22]

To test the impact of baseline symptom severity on the distribution of results, a stratified analysis was performed for the IPSS categories 'moderate symptoms' and 'severe symptoms' because previous research has shown that such stratification has a large impact.[2] No such categories have been defined for the OAB-q SF, so we did not perform a similar analysis for this questionnaire. Subgroup analyses were also performed with participants who received their prescription from their GP, allowing us to provide data that focused on the primary care setting. Finally, we checked if the MID exceeded the measurement error.[15, 17] For this, we calculated the standard error of measurement (SEM) as follows: [SD × (1 − reliability)$^{1/2}$]. Cronbach's alpha was used as the reliability measure.[23]

The complete data set was used without imputing missing data. All analyses were performed using IBM SPSS version 25.0 (IBM Corp., Armonk, NY, USA), and we considered a p-value <0.05 to be statistically significant.

Patient and public involvement

This study was performed without patient involvement. We did not invite patients to comment on the study design nor did we consult them to interpret the results. Patients were not invited to contribute to the writing or editing of this document for readability or accuracy.

RESULTS

For peer review only - http://bmjopen.bmj.com/site/about/guidelines.xhtml

A total of 251 men completed the baseline questionnaires, of which 165 also completed the follow-up questionnaires. The baseline characteristics of men with and without follow-up data are shown in Table 1, with no statistically significant differences found between these groups. Notably, 86.3% of the participants received their prescription from a GP and the remainder received it from a urologist.

Table 1. Baseline characteristics of all participants and participants who dropped out after the baseline questionnaire

	Participants with completed follow-up (n = 165)	Drop out after baseline (n = 86)	p-value
Age (mean ± SD)	66.7±9.7	65.4±12.1	0.42*
Prescription from GP (%)	86.3	83.2	0.55#
IPSS score (mean ± SD)	19.1±6.8	17.6±6.5	0.11*
IPSS categories (%)			0.12#
- none/mild	3.7	6.4	
- moderate	50.9	61.5	
- severe	45.3	32.1	
IPSS Quality of Life (median | IQR)	4.0 | 3.0-5.0	4.0 | 3.0-5.0	0.52§
OAB-q SF (mean ± SD)	39.8±19.2	40.7±18.1	0.70*
Duration of LUTS in months (median | IQR)	24.0 | 5.0-42.0	12.0 | 3.0-36.0	0.11§
History of surgery for LUTS (%)	1.2	3.8	0.19#

IQR: interquartile range. P-values refer to: * student-t-test; # chi-squared test; § Mann–Whitney U test.

Abbreviations: OAB-q SF, Overactive Bladder Questionnaire short form.

There were mean improvements in the IPSS and OAB-q SF scores during the study of 5.8 (SD 6.7) and 11.8 points (SD 17.4), respectively. Between baseline and follow-up at 6 weeks, the mean IPSS score changed from 19.1 (SD 6.8) to 13.3 (SD 6.5) and the mean OAB-q SF score

For peer review only - http://bmjopen.bmj.com/site/about/guidelines.xhtml

changed from 39.7 (SD 19.2) to 27.9 (SD 16.9). The PGI-I outcomes are shown in Table 2 and indicate that most men reported that they were 'a little better' or 'much better' (74.7%), whilst only 23.5% perceived no change. Only three men (1.8%) reported 'worsened' symptoms, and none of the participants reported 'much worsened' or 'very much worsened' symptoms.

Table 2. Change scores for the IPSS and OAB-q SF by PGI-I outcomes

**PGI-I outcome**	**N (%)**	**IPSS**	Missing	**OAB-q SF**	Missing
Very much better	6 (3.6)	13.4 (2.9;23.9)	1	23.8 (2.3;45.3)	0
Much better	50 (30.3)	8.7 (6.8;10.7)	2	19.1 (14.3;24.0)	3
**A little better**	**68 (41.2)**	**5.2 (3.9;6.4)**	**3**	**11.0 (7.1;14.9)**	**4**
No change	38 (23.0)	3.1 (1.1;5.1)	0	3.0 (-2.3;8.4)	4
A little worse	3 (1.8)	-5.0 (-30.9;20.9)	0	-9.7 (-81.7;62.4)	0

Change in IPSS and OAB-q SF scores were estimated by comparing symptom scores between baseline and 6 weeks. Mean change and 95% CIs are presented. Outcomes are inverted so that positive changes reflect symptom improvement. The PGI-I category 'a little better' reflects the MID for both questionnaires. None of the participants scored 'much worsened' or 'very much worsened' on the PGI-I. Abbreviations: MID, Minimal important difference; OAB-q SF, Overactive Bladder Questionnaire short form; PGI-I, Patient Global Impression of Improvement.

Table 2 also shows the distribution of changes in the IPSS and OAB-q SF for each PGI-I category. The MID for the IPSS was 5.2 points (95% CI, 3.9–6.4) and the PGI-I outcomes 'no change' and 'much better' corresponded to IPSS symptom changes of 3.1 points (95% CI, 1.1–5.1) and 8.7 points (95% CI, 6.8–10.7), respectively. The MID for the OAB-q SF was 11.0 points (95% CI, 7.1 to 14.9) and the PGI-I outcomes 'no change' and 'much better' corresponded with mean improvements of 3.0 points (95% CI, -2.3 to 8.4) and 19.1 points (95% CI, 14.3–24.0), respectively. For both questionnaires, the confidence intervals of the MID-categories showed an overlap with the 'no change' categories.

The Spearman correlation coefficients were then calculated between the PGI-I and both

For peer review only - http://bmjopen.bmj.com/site/about/guidelines.xhtml

the IPSS and the OAB-q SF. The correlation was -0.51 for the PGI-I and baseline IPSS, 0.43 for the PGI-I and follow-up IPSS and 0.38 for the PGI-I and change in IPSS. The corresponding correlations for the OAB-q SF were -0.09 at baseline, 0.36 at follow-up and 0.42 for the change.

Table 3. Change scores for the IPSS and OAB-q SF by PGI-I outcomes: subgroup analysis for GP prescriptions

PGI-I outcome	N (%)	IPSS	Missing	OAB-q SF	Missing
Very much better	4 (3.1)	18.0 (1.7;34.3)	1	30.8 (-0.9;62.4)	0
Much better	39 (30.2)	9.2 (7.0;11.5)	2	19.9 (14.8;24.9)	2
**A little better**	**57 (44.2)**	**5.4 (4.0;6.7)**	**3**	**11.2 (7.0;15.4)**	**3**
No change	27 (20.9)	3.1 (0.5;5.6)	0	3.3 (-3.4;9.9)	3
A little worse	2 (1.6)	-8.5 (-11.6.5; 99.5)	0	-16.5 (-353.2;320.2)	0

Change in IPSS and OAB-q SF scores were estimated by comparing symptom scores between baseline and 6 weeks. Mean change and 95% CIs are presented. Outcomes are inverted so that positive changes reflect symptom improvement. The PGI-I category 'a little better' reflects the MID for both questionnaires. None of the participants scored 'much worsened' or 'very much worsened' on the PGI-I. Abbreviations: MID, Minimal important difference; OAB-q SF, Overactive Bladder Questionnaire short form; PGI-I, Patient Global Impression of Improvement.

Subgroup analyses of data for men with a prescription from a GP found no relevant differences, with MID values of 5.4 for the IPSS and 11.2 for the OAB-q SF (Table 3). Stratified analysis of baseline data revealed that men with severe symptoms had higher MID values for the IPSS, reaching 7.1 (95% CI, 5.3–9.0), compared with the MID value of 3.2 (95% CI, 1.7–4.8) for men with moderate symptoms (Table 4).

Table 4. Change scores for the IPSS by PGI-I outcomes

For peer review only - http://bmjopen.bmj.com/site/about/guidelines.xhtml

	Moderate symptoms (n = 88)			Severe symptoms (n = 73)	
**PGI-I outcome**	**N (%)**	**Change**	**Missing**	**N (%)**	**Change**
Very much better	3 (3.4)	6.5 (-50.7;63.7)	1	3 (4.1)	18 (1.7;34.3)
Much better	30 (34.1)	5.6 (3.7;7.5)	1	19 (26.0)	13.5 (10.5;16.5)
**A little better**	33 (37.5)	3.2 (1.7;4.8)	0	32 (43.8)	7.1 (5.3;9.0)
No change	19 (21.6)	1.3 (-1.7;4.3)	0	19 (26.0)	4.9 (2.3;7.6)
A little worse	3 (3.4)	-5.0 (-31.0;20.9)	0	0 (0.0)	–

These results are stratified by baseline symptom severity on the IPSS: moderate symptoms are scores of 8–19 and severe symptoms are scores of ≥20. Change in IPSS scores were estimated by comparing symptom scores between baseline and 6 weeks. Mean change and 95% CIs are presented. Outcomes are inverted so that positive changes reflect symptom improvement. The PGI-I category 'a little better' reflects the MID for both questionnaires. None of the participants scored 'much worsened' or 'very much worsened' on the PGI-I. Abbreviations: MID, Minimal important difference; OAB-q SF, Overactive Bladder Questionnaire short form; PGI-I, Patient Global Impression of Improvement.

Finally, the SEM was 3.6 for the IPSS and 9.7 for the OAB-q SF.

DISCUSSION

We estimated the MID for two questionnaires that are often used to assess male LUTS in primary care. However, whereas the SEM of the IPSS was less than the 95% CI of the MID (5.2 points; 95% CI, 3.9–6.4; SEM 3.6), the SEM of the OAB-q SF fell within the 95% CI of the MID (11 points; 95% CI, 7.1–14.9; SEM 9.7). Thus, we can only conclude that the outcomes for the IPSS were unlikely to have occurred because of chance or measurement imprecision. Given that many questionnaires have used multiple MID values, we were surprised to find only one previous estimate of the MID for the IPSS in the literature. [1, 2] Our study therefore adds relevant information in the primary care setting for clinicians and guideline developers.

Our results for the IPSS were different to those of the seminal study on this topic performed by Barry et al. in secondary care. [1, 2] In that study, the MID of 3.1 points (SD 0.27) fell

For peer review only - http://bmjopen.bmj.com/site/about/guidelines.xhtml

within the 95% CI of the ‘no change’ group (consisting of men who expressed that they hadn’t experienced any change in symptoms), but outside the CI of the ‘a little better’ group, suggesting a likely underestimation of the real value. In the current study, there was also some overlap between the CIs of the ‘no change’ and the ‘a little better’ group, though this was within a change of only 3.9 to 5.1 points. Given that treatment is typically in primary care, we have therefore provided additional data that is applicable to most men with LUTS. Nevertheless, the differences in outcomes compared with the study by Barry et al. need to be explained. It is our contention that three methodological differences account for these differences.

First, Barry et al. compared patients between baseline and follow-up after 13 weeks. By contrast, the follow-up period in the current study was only 6 weeks. Although this difference of 7 weeks may have affected the ability of patients to recall their prior health state accurately, the true impact of this remains unclear. Change scores may also have been influenced by the natural variation that occurs in symptom severity over time.

Second, in the research by Barry et al., a different global assessment of patient improvement was used. This included a 5-point scale with the options ‘marked improvement’, ‘moderate improvement’, ‘slight improvement’, ‘no improvement’, and ‘worse’ for which the exact question was not reported. In our study, we used a 7-point Likert-type scale that ranged from ‘very much better’ to ‘very much worsened’. We considered that this difference probably had no more than a marginal impact given that the positive outcome categories were comparable in both studies. Notably, none of the participants in our study reported that the symptoms had ‘very’ or ‘very much’ worsened.

Third, we mainly included men from primary care, rather than men solely from secondary care. Although it is generally thought that men in primary care have fewer symptoms, our men tended to have more severe symptoms (IPSS score >19) than in the study by Barry et al. (45% versus 25%). Barry et al. also reported that baseline severity had a major impact on the MID, but when we compare their stratified analysis with ours, we had higher mean change scores for each PGI-I category. This might be explained by the fact that we only included men

For peer review only - http://bmjopen.bmj.com/site/about/guidelines.xhtml

who actually used an alpha-blocker. In contrast to this focused approach, Barry et al. used data for all participants in a large, randomized, double-blind trial of four treatment strategies for male LUTS. In their study, a lower MID could therefore have resulted from the inclusion of patients receiving placebo, finasteride, terazosin or combination therapy given that the efficacy of alpha-blockers exceeds that of both placebo and finasteride. The use of blinding meant that men who used placebo or finasteride may have overestimated their subjective improvement, whilst alpha-blocker users may have underestimated their subjective improvement. Given that the IPSS objectively counts symptoms, the placebo and finasteride users would experience a smaller change in the IPSS whereas the active drug users would experience a larger effect. Although the actual impact of each intervention is unknown, researchers in other fields have made similar observations. [10, 11, 24]

We were unable to find any prior estimates of the MID for the OAB-q SF in the literature. Our finding that 11.0 points (95% CI, 7.1–14.9) indicates a clinically relevant change is therefore a novel and important finding, but one for which the reliability will need to be assessed in other studies. We recognize that alpha-blockers are not specifically indicated for the treatment of overactive bladder, but we contend that there is a considerable overlap with LUTS unrelated to overactive bladder. Indeed, guidelines suggest prescribing alpha-blockers for most men with LUTS who request active treatment. This is because these agents have a rapid onset of action, good efficacy, and low rate and severity of adverse events. [3-5] We recommend further study to determine the MID in men with specific symptoms of overactive bladder treated with anticholinergics or beta-3 agonists.

Some limitations need to be considered when assessing our results. Notably, the sample size of this study was small, which resulted in very low numbers of men being included in the PGI-I category 'very much better'. For that category, the mean change scores for both questionnaires showed very wide CIs. The same holds for the categories linked to symptom worsening. In those categories, a discontinuation trial, in which men stop their treatment, may be more suitable for reliable estimates. The sample size might also explain why the CI of the

For peer review only - http://bmjopen.bmj.com/site/about/guidelines.xhtml

MID estimate for the OAB-q SF included the SEM.

Another limitation is reflected by difficulties we encountered with some of the associations between the PGI-I and the two PROM questionnaires. For the IPSS, the follow-up IPSS and PGI-I scores correlated better than with IPSS change and PGI-I scores, suggesting that this rating only reflected the current status, which in turn, decreases confidence in the MID estimate. For the OAB-q SF, the correlation coefficient between the baseline OAB-q SF and PGI-I scores was opposite in magnitude to that for the follow-up OAB-q SF and PGI-I scores. With both questionnaires, the correlation coefficients for the change scores were lower than the threshold of 0.5 that we set a priori.[21] High correlation coefficients are preferred between the anchor and the change in PROM, though some researchers have suggested applying lower thresholds.[25] Still, even the high correlation coefficients are insufficient to confirm that the transition rating is in fact measuring change as opposed to current health status.[21] Unfortunately, Barry et al. did not report the correlation coefficients between the IPSS and the anchor,[2] which is consistent with most other research for PROMs.[26] Given the suboptimal relationship between the PROM and the anchor, we must stress that the estimates obtained for the MID should be interpreted with caution and should be confirmed in future investigations with larger samples.

In conclusion, this study is the first to define MID values for two important PROMs used to evaluate the effectiveness of treatment for male LUTS in primary care. Given that many men are treated in primary care, MID values for this setting are particularly important to inform evidence-based decision-making and to facilitate interpretation of the IPSS and OAB-q SF. Moreover, we consider that this study emphasizes the importance of the MID to individual patients in daily practice. We defined the MID based on the PGI-I outcome 'a little better' in the present study, but patients may expect 'much better' as an outcome when starting therapy. To date, most outcomes of alpha-blocker and other drug treatments for male LUTS have been expressed as the mean IPSS change scores. In the vast majority of studies [3], difference in IPSS changes between active treatment and placebo have approached, but not exceeded, the previously reported MID of 3.1.[1,2] Applying a threshold for improvement of 5.2 points, as

For peer review only

described in our study, may change the interpretation of those studies.

Acknowledgements

We are grateful for the assistance of Tom Vermist in collecting the data. The authors thank all patients and collaborating pharmacies for their participation, as well as Dr Robert Sykes (www.doctored.org.uk) for providing editorial services.

Author contributions

The corresponding author attests that all listed authors meet authorship criteria and that no others meeting the criteria have been omitted.

MHB and MGS initiated the study. Acquisition of the data was done by MHB and MR. Analysis and interpretation of the data was done by MHB, HJA, TD and HW. MHB wrote the manuscript with extensive support from HJA and TD.

All authors critically reviewed the manuscript.

Data sharing statement

Data collected for this study will be available from the corresponding author upon request.

REFERENCES

1 Barry MJ, Fowler FJ,Jr, O'Leary MP, et al. The American Urological Association symptom index for benign prostatic hyperplasia. The Measurement Committee of the American Urological Association. *J Urol* 1992;148:1549,57; discussion 1564.

2 Barry MJ, Williford WO, Chang Y, et al. Benign prostatic hyperplasia specific health status measures in clinical research: how much change in the American Urological Association symptom index and the benign prostatic hyperplasia impact index is perceptible to patients?. *J Urol* 1995;154:1770-4.

3 National Clinical Guideline Centre (NICE). Lower urinary tract symptoms in men: management (Clinical guideline CG97) 2015;CG97 doi:http://www.nice.org.uk/nicemedia/live/12984/48554/48554.pdf;.

4 Gratzke C, Bachmann A, Descazeaud A, et al. EAU Guidelines on the Assessment of Non-neurogenic Male Lower Urinary Tract Symptoms including Benign Prostatic Obstruction. *Eur Urol* 2015;67:1099-109 doi:10.1016/j.eururo.2014.12.038 [doi].

5 Blanker MH, Klomp MA, van den Donk M, et al. Summary of the NHG practice guideline 'Lower urinary tract symptoms in men'. *Ned Tijdschr Geneeskd* 2013;157:A6178.

For peer review only

6 Coyne K, Revicki D, Hunt T, et al. Psychometric validation of an overactive bladder symptom and health-related quality of life questionnaire: the OAB-q. *Qual Life Res* 2002;11:563-74.

7 Coyne KS, Matza LS, Thompson CL. The responsiveness of the Overactive Bladder Questionnaire (OAB-q). *Qual Life Res* 2005;14:849-55.

8 Coyne KS, Thompson CL, Lai JS, et al. An overactive bladder symptom and health-related quality of life short-form: validation of the OAB-q SF. *Neurourol Urodyn* 2015;34:255-63 doi:10.1002/nau.22559 [doi].

9 Jaeschke R, Singer J, Guyatt GH. Measurement of health status. Ascertaining the minimal clinically important difference. *Control Clin Trials* 1989;10:407-15.

10 Terwee CB, Roorda LD, Dekker J, et al. Mind the MIC: large variation among populations and methods. *J Clin Epidemiol* 2010;63:524-34 doi:10.1016/j.jclinepi.2009.08.010 [doi].

11 Olsen MF, Bjerre E, Hansen MD, et al. Minimum clinically important differences in chronic pain vary considerably by baseline pain and methodological factors: systematic review of empirical studies. *J Clin Epidemiol* 2018;101:87,106.e2 doi:S0895-4356(18)30124-0 [pii].

12 van der Worp H, Kollen BJ, Vermist T, et al. Symptom improvement and predictors associated with improvement after 6 weeks of alpha-blocker therapy: An exploratory, single-arm, open-label cohort study. *PLoS One* 2019;14:e0220417 doi:10.1371/journal.pone.0220417 [doi].

13 Viktrup L, Hayes RP, Wang P, et al. Construct validation of patient global impression of severity (PGI-S) and improvement (PGI-I) questionnaires in the treatment of men with lower urinary tract symptoms secondary to benign prostatic hyperplasia. *BMC Urol* 2012;12:30,2490-12-30 doi:10.1186/1471-2490-12-30 [doi].

14 Barendrecht MM, Abrams P, Schumacher H, et al. Do alpha1-adrenoceptor antagonists improve lower urinary tract symptoms by reducing bladder outlet resistance?. *Neurourol Urodyn* 2008;27:226-30 doi:10.1002/nau.20481 [doi].

15 Yalcin I, Bump RC. Validation of two global impression questionnaires for incontinence. *Am J Obstet Gynecol* 2003;189:98-101.

16 Angst F, Aeschlimann A, Angst J. The minimal clinically important difference raised the significance of outcome effects above the statistical level, with methodological implications for future studies. *J Clin Epidemiol* 2017;82:128-36 doi:S0895-4356(16)30776-4 [pii].

17 Crosby RD, Kolotkin RL, Williams GR. Defining clinically meaningful change in health-related quality of life. *J Clin Epidemiol* 2003;56:395-407 doi:S0895435603000441 [pii].

18 Norman GR, Sloan JA, Wyrwich KW. Interpretation of changes in health-related quality of life: the remarkable universality of half a standard deviation. *Med Care* 2003;41:582-92 doi:10.1097/01.MLR.0000062554.74615.4C [doi].

19 de Vet HC, Terwee CB. The minimal detectable change should not replace the minimal important difference. *J Clin Epidemiol* 2010;63:804,5; author reply 806 doi:10.1016/j.jclinepi.2009.12.015 [doi].

20 Cella D, Hahn EA, Dineen K. Meaningful change in cancer-specific quality of life scores: differences between improvement and worsening. *Qual Life Res* 2002;11:207-21.

For peer review only

21 Guyatt GH, Norman GR, Juniper EF, et al. A critical look at transition ratings. *J Clin Epidemiol* 2002;55:900-8 doi:S0895435602004353 [pii].

22 Guyatt GH, Jaeschke RJ. Reassessing quality-of-life instruments in the evaluation of new drugs. *Pharmacoeconomics* 1997;12:621-6 doi:10.2165/00019053-199712060-00002 [doi].

23 Wyrwich KW, Tierney WM, Wolinsky FD. Using the standard error of measurement to identify important changes on the Asthma Quality of Life Questionnaire. *Qual Life Res* 2002;11:1-7.

24 Devji T, Guyatt GH, Lytvyn L, et al. Application of minimal important differences in degenerative knee disease outcomes: a systematic review and case study to inform BMJ Rapid Recommendations. *BMJ Open* 2017;7:e015587,2016-015587 doi:10.1136/bmjopen-2016-015587 [doi].

25 Revicki D, Hays RD, Cella D, et al. Recommended methods for determining responsiveness and minimally important differences for patient-reported outcomes. *J Clin Epidemiol* 2008;61:102-9 doi:10.1016/j.jclinepi.2007.03.012 [doi].

26 Alma H, de Jong C, Tsiligianni I, et al. Clinically relevant differences in COPD health status: systematic review and triangulation. *Eur Respir J* 2018;52:1800412 doi:10.1183/13993003.00412.

For peer review only

INTERNATIONAL-PROSTATE SYMPTOM SCORE (IPSS)

	**Not at all**	**Less than 1 time in 5**	**Less than half the time**	**About half the time**	**More than half the time**	**Almost always**
1. Over the past 4 weeks, how often have you had a sensation of not emptying your bladder completely after you finished urinating?	0	1	2	3	4	5
2. Over the past 4 weeks, how often have you had to urinate again less than two hours after you finished urinating?	0	l	2	3	4	5
3. Over the past 4 weeks, how often have you found you stopped and started again several times when you urinated?	0	1	2	3	4	5
4. Over the past 4 weeks, how often have you found it difficult to postpone urination?	0	1	2	3	4	5
5 Over the past 4 weeks, how often has your urinary stream been weaker than usual?	0	1	2	3	4	5
6 Over the past 4 weeks, how often have you had to push or strain to begin urination?	0	1	2	3	4	5
	None	1 time	2 times	3 times	4 times	5 or more times
7. Over the past 4 weeks, how many times, in general, did you get up to urinate from the time you went to bed at night until the time you got up in the morning?	0	1	2	3	4	5

IPSS Quality of life question

	Delighted	Pleased	Mostly satisfied	Mixed - neither satisfied nor dissatisfied	Mostly dissatisfied	Unhappy	Terrible
If you were to spend the rest of your life with your urinary condition just the way it is now, how would you feel about that?	0	1	2	3	4	5	6

OAB-q-SF

This questionnaire asks about how much you have been bothered by selected bladder symptoms during the past 4 weeks. Please place a ✓ or x in the box that best describes the extent to which you were bothered by each symptom during the past 4 weeks. There are no right or wrong answers. Please be sure to answer every question.

During the past 4 weeks, how bothered were you by...

	Not at all	A little bit	Some-what	Quite a bit	A great deal	A very great deal
1. An uncomfortable urge to urinate?	☐	☐	☐	☐	☐	☐
2. A sudden urge to urinate with little or no warning?	☐	☐	☐	☐	☐	☐
3. Accidental loss of small amounts of urine?	☐	☐	☐	☐	☐	☐
4. Nighttime urination?	☐	☐	☐	☐	☐	☐
5. Waking up at night because you had to urinate?	☐	☐	☐	☐	☐	☐
6. Urine loss associated with a strong desire to urinate?	☐	☐	☐	☐	☐	☐

Patient Global Impression of Improvement, PGI-I

Have your symptoms changed since the start of the medication (the moment you completed the previous questionnaire)?

Very much better	Much better	A little better	No change	A little worse	Much worse	Very much worse
☐	☐	☐	☐	☐	☐	☐

For peer review only

STROBE Statement—Checklist of items that should be included in reports of ***cohort studies***

	Item No	Recommendation	Page No
**Title and abstract**	1	(*a*) Indicate the study's design with a commonly used term in the title or the abstract	2
		(*b*) Provide in the abstract an informative and balanced summary of what was done and what was found	2
**Introduction**			
Background/rationale	2	Explain the scientific background and rationale for the investigation being reported	4
Objectives	3	State specific objectives, including any prespecified hypotheses	4
**Methods**			
Study design	4	Present key elements of study design early in the paper	4-5
Setting	5	Describe the setting, locations, and relevant dates, including periods of recruitment, exposure, follow-up, and data collection	5
Participants	6	(*a*) Give the eligibility criteria, and the sources and methods of selection of participants. Describe methods of follow-up	5
		(*b*) For matched studies, give matching criteria and number of exposed and unexposed	
Variables	7	Clearly define all outcomes, exposures, predictors, potential confounders, and effect modifiers. Give diagnostic criteria, if applicable	5-6
Data sources/ measurement	8*	For each variable of interest, give sources of data and details of methods of assessment (measurement). Describe comparability of assessment methods if there is more than one group	5-6
Bias	9	Describe any efforts to address potential sources of bias	7
Study size	10	Explain how the study size was arrived at	-
Quantitative variables	11	Explain how quantitative variables were handled in the analyses. If applicable, describe which groupings were chosen and why	6
Statistical methods	12	(*a*) Describe all statistical methods, including those used to control for confounding	6-7
		(*b*) Describe any methods used to examine subgroups and interactions	6
		(*c*) Explain how missing data were addressed	7
		(*d*) If applicable, explain how loss to follow-up was addressed	
		(*e*) Describe any sensitivity analyses	
**Results**			
Participants	13*	(a) Report numbers of individuals at each stage of study—eg numbers potentially eligible, examined for eligibility, confirmed eligible, included in the study, completing follow-up, and analysed	7
		(b) Give reasons for non-participation at each stage	
		(c) Consider use of a flow diagram	
Descriptive data	14*	(a) Give characteristics of study participants (eg demographic, clinical, social) and information on exposures and potential confounders	8
		(b) Indicate number of participants with missing data for each variable of interest	8
		(c) Summarise follow-up time (eg, average and total amount)	8
Outcome data	15*	Report numbers of outcome events or summary measures over time	-

For peer review only

Main results	16	(*a*) Give unadjusted estimates and, if applicable, confounder-adjusted estimates and their precision (eg, 95% confidence interval). Make clear which confounders were adjusted for and why they were included	7-11
		(*b*) Report category boundaries when continuous variables were categorized	7-11
		(*c*) If relevant, consider translating estimates of relative risk into absolute risk for a meaningful time period	
Other analyses	17	Report other analyses done—eg analyses of subgroups and interactions, and sensitivity analyses	7-11
**Discussion**			
Key results	18	Summarise key results with reference to study objectives	11
Limitations	19	Discuss limitations of the study, taking into account sources of potential bias or imprecision. Discuss both direction and magnitude of any potential bias	13-14
Interpretation	20	Give a cautious overall interpretation of results considering objectives, limitations, multiplicity of analyses, results from similar studies, and other relevant evidence	11-14
Generalisability	21	Discuss the generalisability (external validity) of the study results	11
**Other information**			
Funding	22	Give the source of funding and the role of the funders for the present study and, if applicable, for the original study on which the present article is based	3

*Give information separately for exposed and unexposed groups.

Note: An Explanation and Elaboration article discusses each checklist item and gives methodological background and published examples of transparent reporting. The STROBE checklist is best used in conjunction with this article (freely available on the Web sites of PLoS Medicine at http://www.plosmedicine.org/, Annals of Internal Medicine at http://www.annals.org/, and Epidemiology at http://www.epidem.com/). Information on the STROBE Initiative is available at http://www.strobe-statement.org.

For peer review only

BMJ Open

Determining the minimal important differences in the International Prostate Symptom Score and Overactive Bladder Questionnaire: results from an observational cohort study in Dutch primary care

Journal:	*BMJ Open*
Manuscript ID	bmjopen-2019-032795.R2
Article Type:	Original research
Date Submitted by the Author:	25-Nov-2019
Complete List of Authors:	Blanker, Marco; University of Groningen, University Medical Center Groningen, department of General Practice and Elderly Care Medicine<br>Alma, Harma; University of Groningen, University Medical Center Groningen, Department of General Practice and Elderly Care Medicine; University of Groningen, University Medical Center Groningen, Groningen Research Institute for Asthma and COPD (GRIAC)<br>Devji, Tahira; McMaster University Faculty of Health Sciences, Clinical Epidemiology and Biostatistics<br>Roelofs, Marjan; University of Groningen, University Medical Center Groningen, department of General Practice and Elderly Care Medicine<br>Steffens, Martijn ; Isala Hospitals, urology<br>van der Worp, Henk; University of Groningen, University medical center groningen, Department General Practice and Elderly Care Medicine
**Primary Subject Heading**:	General practice / Family practice
Secondary Subject Heading:	Urology, Epidemiology
Keywords:	Lower Urinary Tract Symptoms, Adrenergic alpha-Antagonists, Primary Health Care, Minimal Clinically Important Difference, Patient outcome assessment

SCHOLARONE™
Manuscripts

Determining the minimal important differences in the International Prostate Symptom Score and Overactive Bladder Questionnaire: results from an observational cohort study in Dutch primary care

M.H. (Marco) Blanker[1], H.J. (Harma) Alma[1], T. (Tahira) Devji[2], M. (Marjan) Roelofs[1], M.G. (Martijn) Steffens[3], H. (Henk) van der Worp[1]

1. Department of General Practice and Elderly Care Medicine, University Medical Centre Groningen, University of Groningen
2. Department of Health Research Methods, Evidence and Impact, McMaster University, Canada
3. Department of Urology, Isala Clinics, Zwolle, The Netherlands

Corresponding author: Marco H. Blanker, MD PhD, general practitioner and epidemiologist, Department of General Practice and Elderly Care Medicine, University Medical Centre Groningen, University of Groningen, P.O. box 196, 9700 AD Groningen, The Netherlands, Phone: +31 6 14961542, Fax: +31 50 3632964

E-mail addresses: m.h.blanker@umcg.nl, h.j.alma@umcg.nl , devjits@mcmaster.ca , marjanroelofs@hotmail.com , m.steffens@isala.nl , h.van.der.worp@umcg.nl

Wordcount: 3,116

Keywords: Lower Urinary Tract Symptoms; Adrenergic alpha-Antagonists; Primary Health Care; Minimal Clinically Important Difference; Patient outcome assessment

For peer review only

ABSTRACT

Objectives: To determine the minimal important difference (MID) of the International Prostate Symptom Score (IPSS) and the Overactive Bladder Questionnaire short form (OAB-q SF) assessed in primary care among patients treated for lower urinary tract symptoms (LUTS).

Design: Single-arm, open-label observational cohort study with a 6-week follow-up.

Setting: Twenty-two pharmacies in the Netherlands.

Participants: We enrolled Dutch men with uncomplicated LUTS who received a new alpha-blocker prescription from their general practitioner or urologist.

Primary and secondary outcomes: The IPSS and OAB-q SF were completed before and after 6 weeks of therapy. At 6 weeks, men also completed the Patient Global Impression of Improvement (PGI-I). The mean change scores of the IPSS and OAB-q SF were calculated for each PGI-I outcome category, with the category 'a little better' used to determine the MID. The standard error of measurement (SEM) was calculated for each questionnaire.

Results: In total, 165 men completed follow-up. The MID was 5.2 points (95%CI, 3.9–6.4; SEM 3.6) for the IPSS and 11.0 points (95%CI, 7.1–14.9; SEM 9.7) for the OAB-q SF. For both questionnaires, confidence intervals showed an overlap with the no-change categories. However, the MID for the IPSS was higher in men with severe baseline symptoms (7.1; 95%CI, 5.3–9.0) than in men with moderate baseline symptoms (3.2; 95%CI, 1.7–4.8).

Conclusion: In this study, the MID for the IPSS was considerably higher than the MID of 3.1 reported in the only other study on this topic, but may be due to methodological differences. Interpretation of the MID for the OAB-q SF is hampered by the overlap with the SEM. Future studies are needed to confirm our results because correlations between the PGI-I and symptom questionnaires were suboptimal.

ARTICLE SUMMARY

Strengths and limitations of this study:

- We assessed the minimal important difference (MID) of two frequently used questionnaires

For peer review only

on lower urinary tract symptoms.

- Given that many men are treated in primary care, MID values for this setting are particularly important to inform evidence-based decision-making and to facilitate interpretation of the IPSS and OAB-q SF.
- Notably, the sample size of this study was small, which resulted in very low numbers of men being included in the PGI-I category 'very much better' or 'worsening of symptoms', and may clarify the small overlap of the confidence intervals with the no-change category.

Funding statement: The Hein Hogerzeil Foundation supported this work with an unrestricted grant. The Foundation was not involved in the design of this study, nor in the data collection, data analyses, interpretation of the outcomes, or the writing of this manuscript.

Conflict of interest statement: All authors have completed the ICMJE uniform disclosure form at www.icmje.org/coi_disclosure.pdf and declare: no support from any organisation for the submitted work; no financial relationships with any organisations that might have an interest in the submitted work in the previous three years; no other relationships or activities that could appear to have influenced the submitted work.

For peer review only

INTRODUCTION

Symptom severity is a key outcome for patients with lower urinary tract symptoms (LUTS) and is most often evaluated by direct patient inquiry, using patient-reported outcome measures (PROMs). Although the IPSS is most often used for this purpose in both clinical trials and practice,[1-5] it fails to capture problematic symptoms such as urinary incontinence and urgency. Therefore, the Overactive Bladder Questionnaire (OAB-q) is increasingly being used to evaluate the treatment of overactive bladder,[6, 7] with the short form (i.e., OAB-q SF) having the advantage of being less time consuming.[8] Together, both of the IPSS and OAB-q SF capture the spectrum of outcomes that are important to patients, but it is difficult to interpret the effects of an intervention expressed as mean scores or change scores over time.

The minimal important difference (MID) has proven invaluable when interpreting PROMs and could be of great value for both the IPSS and OAB-q SF.[9, 10] To date, the MID has only been reported for the IPSS in a study conducted in secondary care among participants of clinical trials.[1, 2] There has been no report of the MID for the OAB-q SF in any care setting. In countries like the Netherlands and the United Kingdom, most men with LUTS first visit their GP to seek treatment. Given that setting may affect the MID, possibly because of differences in baseline symptom severity,[9-11] we feel that it is important to assess the MID in a primary care setting. To date the MID for secondary care settings has been applied in guidelines for primary care.[3, 5] It is unclear if applying the threshold for a clinically relevant outcome is appropriate. Men who receive treatment need to be aware of what can be expected. Knowledge about the MID in primary care will then provide invaluable data for interpreting treatment outcomes that may differ between primary and secondary care. In addition, evidence must be obtained from multiple studies to ensure that MID determinations are accurate.

In the current study, we aimed to determine the MIDs for both the IPSS and OAB-q SF in a patient cohort originating mainly from primary care.

METHODS

For peer review only - http://bmjopen.bmj.com/site/about/guidelines.xhtml

Study design

We conducted a prospective cohort study between January 2016 and April 2018.[12] Baseline data for the IPSS and OAB-q SF were compared with follow-up data after 6 weeks of treatment. At follow-up, participants also completed the Patient Global Impression of Improvement (PGI-I) and we calculated the MID.

Participants

Adult men who visited a participating pharmacy in the north of the Netherlands were included if they received a new alpha-blocker prescription for uncomplicated LUTS from a GP or urologist. A prescription was defined as new if no alpha-blocker prescription had been given within the past year. The pharmacists checked if the alpha-blocker was indicated for LUTS and excluded men prescribed alpha-blockers for urinary tract stones or indwelling catheters. All participants provided written informed consent. The medical ethics committee of the University Medical Centre Groningen, the Netherlands, approved the study (number 2016.122).

Data collection

At baseline, before starting alpha-blocker therapy, all participants provided relevant descriptive data (e.g., age, duration of LUTS in months or years, and history of surgery for LUTS) and completed the Dutch versions of the IPSS and OAB-q SF. After 6 weeks, men who consented repeated the IPSS and OAB-q SF by postal invitation. At this time, we asked participants to complete the Patient Global Impression of Improvement (PGI-I) questionnaire.[13] The period of six weeks was chosen as clinical effects of alpha-blockers take a few weeks to develop fully, but significant efficacy over placebo can occur within hours to days.[4, 14]

Questionnaires

The IPSS questionnaire was originally validated as the *American Urological Association Symptom Index for benign prostatic hyperplasia*.[1] It includes 7 questions covering frequency, nocturia, weak urinary stream, hesitancy, intermittence, incomplete emptying and urgency. Each question has response options ranging from 0 to five, with higher scores reflecting more severe symptoms. Total scores that may range from 0 (no symptoms) to 35 points (maximum score),

For peer review only

and scores are often categorized as no/mild symptoms (0–7 points), moderate symptoms (8–19 points), or severe symptoms (≥20 points). The questionnaire was internally consistent (Cronbach's alpha = 0.86) and has excellent test-retest reliability (r = 0,92).[1] The MID for the IPSS is currently considered to be 3.1 points.[2] The AUA-SI has been internationally adopted and implemented worldwide under the name IPSS.

IPSS focuses on the concept of "benign prostatic hyperplasia" as cause of male LUTS, which appeared to have a multifactorial origin. Overactive bladder (OAB) is one of the alternative explanations of LUTS. Although urgency (included in the IPSS) relates to OAB, OAB includes other symptoms as well, which are not included in the IPSS questionnaire. Therefor, Coyne et all developed a condition specific questionnaire, the OAB-q.[6, 7] The OAB-q was developed from focus groups of men and women, clinician opinion, and a thorough literature review. More recently, this OAB-q has been shortened to benefit patients, researchers and clinicians.[8] The OAB-q SF contains six questions on 6-point Likert-type scales, with the outcomes transformed to a 0–100 point scale in which higher scores indicate more severe symptoms.[8] This scale demonstrated good convergent validity, discriminant validity, internal reliability, reproducibility, and responsiveness to change.[8]

Both IPSS and OAB-q-SF capture symptoms that are not by definition patient important, but rather reflect the conditions under study. To study if changes on a questionnaire over time are relevant for patients, the PGI-I has been developed using a quantitative approach.[13, 15]

The PGI-I is a validated generic tool for assessing overall improvement after treatment and is answered on a 7-point Likert-type scale, with the following options: 'very much better', 'much better', 'a little better', 'no change', 'a little worse', 'much worse' or 'very much worse'.[13, 15] Full versions of these questionnaires are presented as supplementary file 1.

We sent a reminder after two weeks to patients who did not respond to follow-up requests.

Data analyses

Baseline characteristics are reported as continuous variables and summarized as mean and SD or as median and interquartile range, depending on the distribution checked by the Shapiro–

For peer review only - http://bmjopen.bmj.com/site/about/guidelines.xhtml

Wilk test. These characteristics were also compared between men with and without completed follow-up data to test for selective nonresponse. Next, the change scores of the IPSS and OAB-q SF were calculated by comparing the data between the baseline and follow-up questionnaires. Change scores were inverted to facilitate intuitive interpretation, with positive scores reflecting symptom improvement.

Various methods exist to determine the MID of questionnaires and are typically either anchor-based or distribution-based.[9, 16-18] The latter involve evaluating change in the PROM with the probability that the change occurred by chance, sample variation or measurement precision; however, they do not reflect patient perspectives.[17, 19] Thus, we used an anchor-based method,[9] in which we compared changes in the IPSS or OAB-q SF (PROM) with the PGI-I (the anchor). For each PGI-I category, we then present the mean change in scores from baseline to follow-up with the associated CIs. We defined the MID as the mean change in IPSS or OAB-q SF for the PGI-I category 'a little better', as the M in MID reflects the *minimal* change that is considered relevant. We also present the mean change scores for the other PGI-I categories.

The usefulness of anchor-based approaches depends on the relationship between the PROM and the anchor.[20-22] The anchor and PROM should be measuring the same or similar underlying constructs and should therefore be appreciably correlated. Correlations between questionnaire change scores and the anchor PGI-I should be obviously strong, as else these measure different concepts. Correlations between the anchor PGI-I and the baseline and follow-up questionnaire scores are performed to check for a possible response shift. Mostly anchor PGI-I scores seem correlated with follow-up scores (due to response shift). We therefore examined the Spearman correlation coefficients between the PGI-I and the IPSS and OAB-q SF for the baseline, follow-up and change data to ensure the anchor's validity. A correlation coefficient between the symptom change scores and the PGI-I of ≥0.50, and an equal and opposite correlation of the PGI-I with the baseline score and the follow-up score, were considered ideal and likely to yield trustworthy MID estimates.[20-22]

To test the impact of baseline symptom severity on the distribution of results, a

For peer review only

stratified analysis was performed for the IPSS categories ‘moderate symptoms’ and ‘severe symptoms’ because previous research has shown that such stratification has a large impact.[2] No such categories have been defined for the OAB-q SF, so we did not perform a similar analysis for this questionnaire. Subgroup analyses were also performed with participants who received their prescription from their GP, allowing us to provide data that focused on the primary care setting. Finally, we checked if the MID exceeded the measurement error.[15, 17] For this, we calculated the standard error of measurement (SEM) as follows: $[SD \times (1 - \text{reliability})^{1/2}]$. Cronbach’s alpha was used as the reliability measure.[23]

The complete data set was used without imputing missing data. All analyses were performed using IBM SPSS version 25.0 (IBM Corp., Armonk, NY, USA), and we considered a p-value <0.05 to be statistically significant.

Patient and public involvement

This study was performed without patient involvement. We did not invite patients to comment on the study design nor did we consult them to interpret the results. Patients were not invited to contribute to the writing or editing of this document for readability or accuracy.

RESULTS

A total of 251 men completed the baseline questionnaires, of which 165 also completed the follow-up questionnaires. The baseline characteristics of men with and without follow-up data are shown in Table 1, with no statistically significant differences found between these groups. Notably, 86.3% of the participants received their prescription from a GP and the remainder received it from a urologist.

Table 1. Baseline characteristics of all participants and participants who dropped out after the baseline questionnaire

	Participants with completed follow-up	Drop out after baseline (n = 86)	p-value

For peer review only

	(n = 165)		
Age (mean ± SD)	66.7±9.7	65.4±12.1	0.42*
Prescription from GP (%)	86.3	83.2	0.55#
IPSS score (mean ± SD)	19.1±6.8	17.6±6.5	0.11*
IPSS categories (%)			0.12#
- none/mild	3.7	6.4	
- moderate	50.9	61.5	
- severe	45.3	32.1	
IPSS Quality of Life (median | IQR)	4.0 | 3.0-5.0	4.0 | 3.0-5.0	0.52§
OAB-q SF (mean ± SD)	39.8±19.2	40.7±18.1	0.70*
Duration of LUTS in months (median | IQR)	24.0 | 5.0-42.0	12.0 | 3.0-36.0	0.11§
History of surgery for LUTS (%)	1.2	3.8	0.19#

IQR: interquartile range. P-values refer to: * student-t-test; # chi-squared test; § Mann–Whitney U test.

Abbreviations: OAB-q SF, Overactive Bladder Questionnaire short form.

There were mean improvements in the IPSS and OAB-q SF scores during the study of 5.8 (SD 6.7) and 11.8 points (SD 17.4), respectively. Between baseline and follow-up at 6 weeks, the mean IPSS score changed from 19.1 (SD 6.8) to 13.3 (SD 6.5) and the mean OAB-q SF score changed from 39.7 (SD 19.2) to 27.9 (SD 16.9). The PGI-I outcomes are shown in Table 2 and indicate that most men reported that they were 'a little better' or 'much better' (74.7%), whilst only 23.5% perceived no change. Only three men (1.8%) reported 'worsened' symptoms, and none of the participants reported 'much worsened' or 'very much worsened' symptoms.

Table 2. Change scores for the IPSS and OAB-q SF by PGI-I outcomes

**PGI-I outcome**	**N (%)**	**IPSS**	Missing	**OAB-q SF**	Missing
Very much better	6 (3.6)	13.4 (2.9;23.9)	1	23.8 (2.3;45.3)	0
Much better	50 (30.3)	8.7 (6.8;10.7)	2	19.1 (14.3;24.0)	3

For peer review only - http://bmjopen.bmj.com/site/about/guidelines.xhtml

For peer review only

**A little better**	**68 (41.2)**	**5.2 (3.9;6.4)**	**3**	**11.0 (7.1;14.9)**	**4**
No change	38 (23.0)	3.1 (1.1;5.1)	0	3.0 (-2.3;8.4)	4
A little worse	3 (1.8)	-5.0 (-30.9;20.9)	0	-9.7 (-81.7;62.4)	0

Change in IPSS and OAB-q SF scores were estimated by comparing symptom scores between baseline and 6 weeks. Mean change and 95% CIs are presented. Outcomes are inverted so that positive changes reflect symptom improvement. The PGI-I category 'a little better' reflects the MID for both questionnaires. None of the participants scored 'much worsened' or 'very much worsened' on the PGI-I. Abbreviations: MID, Minimal important difference; OAB-q SF, Overactive Bladder Questionnaire short form; PGI-I, Patient Global Impression of Improvement.

Table 2 also shows the distribution of changes in the IPSS and OAB-q SF for each PGI-I category. The MID for the IPSS was 5.2 points (95% CI, 3.9–6.4) and the PGI-I outcomes 'no change' and 'much better' corresponded to IPSS symptom changes of 3.1 points (95% CI, 1.1–5.1) and 8.7 points (95% CI, 6.8–10.7), respectively. The MID for the OAB-q SF was 11.0 points (95% CI, 7.1 to 14.9) and the PGI-I outcomes 'no change' and 'much better' corresponded with mean improvements of 3.0 points (95% CI, -2.3 to 8.4) and 19.1 points (95% CI, 14.3–24.0), respectively. For both questionnaires, the confidence intervals of the MID-categories showed an overlap with the 'no change' categories.

The Spearman correlation coefficients were then calculated between the PGI-I and both the IPSS and the OAB-q SF. The correlation was -0.51 for the PGI-I and baseline IPSS, 0.43 for the PGI-I and follow-up IPSS and 0.38 for the PGI-I and change in IPSS. The corresponding correlations for the OAB-q SF were -0.09 at baseline, 0.36 at follow-up and 0.42 for the change.

Table 3. Change scores for the IPSS and OAB-q SF by PGI-I outcomes: subgroup analysis for GP prescriptions

**PGI-I outcome**	**N (%)**	**IPSS**	**Missing**	**OAB-q SF**	**Missing**

For peer review only - http://bmjopen.bmj.com/site/about/guidelines.xhtml

Very much better	4 (3.1)	18.0 (1.7;34.3)	1	30.8 (-0.9;62.4)	0
Much better	39 (30.2)	9.2 (7.0;11.5)	2	19.9 (14.8;24.9)	2
**A little better**	**57 (44.2)**	**5.4 (4.0;6.7)**	**3**	**11.2 (7.0;15.4)**	**3**
No change	27 (20.9)	3.1 (0.5;5.6)	0	3.3 (-3.4;9.9)	3
A little worse	2 (1.6)	-8.5 (-11.6.5; 99.5)	0	-16.5 (-353.2;320.2)	0

Change in IPSS and OAB-q SF scores were estimated by comparing symptom scores between baseline and 6 weeks. Mean change and 95% CIs are presented. Outcomes are inverted so that positive changes reflect symptom improvement. The PGI-I category 'a little better' reflects the MID for both questionnaires. None of the participants scored 'much worsened' or 'very much worsened' on the PGI-I. Abbreviations: MID, Minimal important difference; OAB-q SF, Overactive Bladder Questionnaire short form; PGI-I, Patient Global Impression of Improvement.

Subgroup analyses of data for men with a prescription from a GP found no relevant differences, with MID values of 5.4 for the IPSS and 11.2 for the OAB-q SF (Table 3). Stratified analysis of baseline data revealed that men with severe symptoms had higher MID values for the IPSS, reaching 7.1 (95% CI, 5.3–9.0), compared with the MID value of 3.2 (95% CI, 1.7–4.8) for men with moderate symptoms (Table 4).

Table 4. Change scores for the IPSS by PGI-I outcomes

	**Moderate symptoms (n = 88)**			**Severe symptoms (n = 73)**	
**PGI-I outcome**	**N (%)**	**Change**	**Missing**	**N (%)**	**Change**
Very much better	3 (3.4)	6.5 (-50.7;63.7)	1	3 (4.1)	18 (1.7;34.3)
Much better	30 (34.1)	5.6 (3.7;7.5)	1	19 (26.0)	13.5 (10.5;16.5)
**A little better**	33 (37.5)	3.2 (1.7;4.8)	0	32 (43.8)	7.1 (5.3;9.0)
No change	19 (21.6)	1.3 (-1.7;4.3)	0	19 (26.0)	4.9 (2.3;7.6)
A little worse	3 (3.4)	-5.0 (-31.0;20.9)	0	0 (0.0)	–

These results are stratified by baseline symptom severity on the IPSS: moderate symptoms are scores of 8–19 and severe symptoms are scores of ≥20. Change in IPSS scores were estimated by comparing

For peer review only - http://bmjopen.bmj.com/site/about/guidelines.xhtml

symptom scores between baseline and 6 weeks. Mean change and 95% CIs are presented. Outcomes are inverted so that positive changes reflect symptom improvement. The PGI-I category 'a little better' reflects the MID for both questionnaires. None of the participants scored 'much worsened' or 'very much worsened' on the PGI-I. Abbreviations: MID, Minimal important difference; OAB-q SF, Overactive Bladder Questionnaire short form; PGI-I, Patient Global Impression of Improvement.

Finally, the SEM was 3.6 for the IPSS and 9.7 for the OAB-q SF.

For peer review only

DISCUSSION

We estimated the MID for two questionnaires that are often used to assess male LUTS in primary care. However, whereas the SEM of the IPSS was less than the 95% CI of the MID (5.2 points; 95% CI, 3.9–6.4; SEM 3.6), the SEM of the OAB-q SF fell within the 95% CI of the MID (11 points; 95% CI, 7.1–14.9; SEM 9.7). Thus, we can only conclude that the outcomes for the IPSS were unlikely to have occurred because of chance or measurement imprecision. Given that many questionnaires have used multiple MID values, we were surprised to find only one previous estimate of the MID for the IPSS in the literature.[1, 2] Our study therefore adds relevant information in the primary care setting for clinicians and guideline developers.

Our results for the IPSS were different to those of the seminal study on this topic performed by Barry et al. in secondary care.[1, 2] In that study, the MID of 3.1 points (SD 0.27) fell within the 95% CI of the 'no change' group (consisting of men who expressed that they hadn't experienced any change in symptoms), but outside the CI of the 'a little better' group, suggesting a likely underestimation of the real value. In the current study, there was also some overlap between the CIs of the 'no change' and the 'a little better' group, though this was within a change of only 3.9 to 5.1 points. This could be explained by the relative small samples in the subgroup analyses. Given that treatment is typically in primary care, we have therefore provided additional data that is applicable to most men with LUTS. Nevertheless, the differences in outcomes compared with the study by Barry et al. need to be explained. It is our contention that three methodological differences account for these differences.

For peer review only - http://bmjopen.bmj.com/site/about/guidelines.xhtml

First, Barry et al. compared patients between baseline and follow-up after 13 weeks. By contrast, the follow-up period in the current study was only 6 weeks. Although this difference of 7 weeks may have affected the ability of patients to recall their prior health state accurately, the true impact of this remains unclear. Change scores may also have been influenced by the natural variation that occurs in symptom severity over time.

Second, in the research by Barry et al., a different global assessment of patient improvement was used. This included a 5-point scale with the options 'marked improvement', 'moderate improvement', 'slight improvement', 'no improvement', and 'worse' for which the exact question was not reported. In our study, we used a 7-point Likert-type scale that ranged from 'very much better' to 'very much worsened'. We considered that this difference probably had no more than a marginal impact given that the positive outcome categories were comparable in both studies. Notably, none of the participants in our study reported that the symptoms had 'very' or 'very much' worsened.

Third, we mainly included men from primary care, rather than men solely from secondary care. Although it is generally thought that men in primary care have fewer symptoms, our men tended to have more severe symptoms (IPSS score >19) than in the study by Barry et al. (45% versus 25%). Barry et al. also reported that baseline severity had a major impact on the MID, but when we compare their stratified analysis with ours, we had higher mean change scores for each PGI-I category. This might be explained by the fact that we only included men who actually used an alpha-blocker. In contrast to this focused approach, Barry et al. used data for all participants in a large, randomized, double-blind trial of four treatment strategies for male LUTS. In their study, a lower MID could therefore have resulted from the inclusion of patients receiving placebo, finasteride, terazosin or combination therapy given that the efficacy of alpha-blockers exceeds that of both placebo and finasteride. The use of blinding meant that men who used placebo or finasteride may have overestimated their subjective improvement, whilst alpha-blocker users may have underestimated their subjective improvement. Given that the IPSS objectively counts symptoms, the placebo and finasteride users would experience a

For peer review only

smaller change in the IPSS whereas the active drug users would experience a larger effect. Although the actual impact of each intervention is unknown, researchers in other fields have made similar observations.[10, 11, 24]

We were unable to find any prior estimates of the MID for the OAB-q SF in the literature. Our finding that 11.0 points (95% CI, 7.1–14.9) indicates a clinically relevant change is therefore a novel and important finding, but one for which the reliability will need to be assessed in other studies. We recognize that alpha-blockers are not specifically indicated for the treatment of overactive bladder, but we contend that there is a considerable overlap with LUTS unrelated to overactive bladder. Indeed, guidelines suggest prescribing alpha-blockers for most men with LUTS who request active treatment. This is because these agents have a rapid onset of action, good efficacy, and low rate and severity of adverse events.[3-5] We recommend further study to determine the MID in men with specific symptoms of overactive bladder treated with anticholinergics or beta-3 agonists.

Some limitations need to be considered when assessing our results. Notably, the sample size of this study was small, which resulted in very low numbers of men being included in the PGI-I category 'very much better'. For that category, the mean change scores for both questionnaires showed very wide CIs. The same holds for the categories linked to symptom worsening. In those categories, a discontinuation trial, in which men stop their treatment, may be more suitable for reliable estimates. The sample size might also explain why the CI of the MID estimate for the OAB-q SF included the SEM.

Another limitation is reflected by difficulties we encountered with some of the associations between the PGI-I and the two PROM questionnaires. For the IPSS, the follow-up IPSS and PGI-I scores correlated better than with IPSS change and PGI-I scores, suggesting that this rating only reflected the current status, which in turn, decreases confidence in the MID estimate. For the OAB-q SF, the correlation coefficient between the baseline OAB-q SF and PGI-I scores was opposite in magnitude to that for the follow-up OAB-q SF and PGI-I scores. With both questionnaires, the correlation coefficients for the change scores were lower than the threshold

For peer review only

of 0.5 that we set a priori.[21] High correlation coefficients are preferred between the anchor and the change in PROM, though some researchers have suggested applying lower thresholds.[25] Still, even the high correlation coefficients are insufficient to confirm that the transition rating is in fact measuring change as opposed to current health status.[21] Unfortunately, Barry et al. did not report the correlation coefficients between the IPSS and the anchor,[2] which is consistent with most other research for PROMs.[26] Given the suboptimal relationship between the PROM and the anchor, we must stress that the estimates obtained for the MID should be interpreted with caution and should be confirmed in future investigations with larger samples.

In conclusion, this study is the first to define MID values for two important PROMs used to evaluate the effectiveness of treatment for male LUTS in primary care. Given that many men are treated in primary care, MID values for this setting are particularly important to inform evidence-based decision-making and to facilitate interpretation of the IPSS and OAB-q SF. Moreover, we consider that this study emphasizes the importance of the MID to individual patients in daily practice. We defined the MID based on the PGI-I outcome 'a little better' in the present study, but patients may expect 'much better' as an outcome when starting therapy. To date, most outcomes of alpha-blocker and other drug treatments for male LUTS have been expressed as the mean IPSS change scores. In the vast majority of studies [3], difference in IPSS changes between active treatment and placebo have approached, but not exceeded, the previously reported MID of 3.1.[1, 2] Applying a threshold for improvement of 5.2 points, as described in our study, may change the interpretation of those studies.

Acknowledgements

We are grateful for the assistance of Tom Vermist in collecting the data. The authors thank all patients and collaborating pharmacies for their participation, as well as Dr Robert Sykes (www.doctored.org.uk) for providing editorial services.

Author contributions

The corresponding author attests that all listed authors meet authorship criteria and that no

For peer review only - http://bmjopen.bmj.com/site/about/guidelines.xhtml

others meeting the criteria have been omitted.

MHB and MGS initiated the study. Acquisition of the data was done by MHB and MR. Analysis and interpretation of the data was done by MHB, HJA, TD and HW. MHB wrote the manuscript with extensive support from HJA and TD.

All authors critically reviewed the manuscript.

Data sharing statement

Data collected for this study will be available from the corresponding author upon request.

REFERENCES

1 Barry MJ, Fowler FJ,Jr, O'Leary MP, et al. The American Urological Association symptom index for benign prostatic hyperplasia. The Measurement Committee of the American Urological Association. *J Urol* 1992;148:1549,57; discussion 1564.

2 Barry MJ, Williford WO, Chang Y, et al. Benign prostatic hyperplasia specific health status measures in clinical research: how much change in the American Urological Association symptom index and the benign prostatic hyperplasia impact index is perceptible to patients?. *J Urol* 1995;154:1770-4.

3 National Clinical Guideline Centre (NICE). Lower urinary tract symptoms in men: management (Clinical guideline CG97) 2015;CG97 doi:http://www.nice.org.uk/nicemedia/live/12984/48554/48554.pdf;.

4 Gratzke C, Bachmann A, Descazeaud A, et al. EAU Guidelines on the Assessment of Non-neurogenic Male Lower Urinary Tract Symptoms including Benign Prostatic Obstruction. *Eur Urol* 2015;67:1099-109 doi:10.1016/j.eururo.2014.12.038 [doi].

5 Blanker MH, Klomp MA, van den Donk M, et al. Summary of the NHG practice guideline 'Lower urinary tract symptoms in men'. *Ned Tijdschr Geneeskd* 2013;157:A6178.

6 Coyne K, Revicki D, Hunt T, et al. Psychometric validation of an overactive bladder symptom and health-related quality of life questionnaire: the OAB-q. *Qual Life Res* 2002;11:563-74.

7 Coyne KS, Matza LS, Thompson CL. The responsiveness of the Overactive Bladder Questionnaire (OAB-q). *Qual Life Res* 2005;14:849-55.

8 Coyne KS, Thompson CL, Lai JS, et al. An overactive bladder symptom and health-related quality of life short-form: validation of the OAB-q SF. *Neurourol Urodyn* 2015;34:255-63 doi:10.1002/nau.22559 [doi].

9 Jaeschke R, Singer J, Guyatt GH. Measurement of health status. Ascertaining the minimal clinically important difference. *Control Clin Trials* 1989;10:407-15.

10 Terwee CB, Roorda LD, Dekker J, et al. Mind the MIC: large variation among populations and methods. *J Clin Epidemiol* 2010;63:524-34 doi:10.1016/j.jclinepi.2009.08.010 [doi].

For peer review only

11 Olsen MF, Bjerre E, Hansen MD, et al. Minimum clinically important differences in chronic pain vary considerably by baseline pain and methodological factors: systematic review of empirical studies. *J Clin Epidemiol* 2018;101:87,106.e2 doi:S0895-4356(18)30124-0 [pii].

12 van der Worp H, Kollen BJ, Vermist T, et al. Symptom improvement and predictors associated with improvement after 6 weeks of alpha-blocker therapy: An exploratory, single-arm, open-label cohort study. *PLoS One* 2019;14:e0220417 doi:10.1371/journal.pone.0220417 [doi].

13 Viktrup L, Hayes RP, Wang P, et al. Construct validation of patient global impression of severity (PGI-S) and improvement (PGI-I) questionnaires in the treatment of men with lower urinary tract symptoms secondary to benign prostatic hyperplasia. *BMC Urol* 2012;12:30,2490-12-30 doi:10.1186/1471-2490-12-30 [doi].

14 Barendrecht MM, Abrams P, Schumacher H, et al. Do alpha1-adrenoceptor antagonists improve lower urinary tract symptoms by reducing bladder outlet resistance?. *Neurourol Urodyn* 2008;27:226-30 doi:10.1002/nau.20481 [doi].

15 Yalcin I, Bump RC. Validation of two global impression questionnaires for incontinence. *Am J Obstet Gynecol* 2003;189:98-101.

16 Angst F, Aeschlimann A, Angst J. The minimal clinically important difference raised the significance of outcome effects above the statistical level, with methodological implications for future studies. *J Clin Epidemiol* 2017;82:128-36 doi:S0895-4356(16)30776-4 [pii].

17 Crosby RD, Kolotkin RL, Williams GR. Defining clinically meaningful change in health-related quality of life. *J Clin Epidemiol* 2003;56:395-407 doi:S0895435603000441 [pii].

18 Norman GR, Sloan JA, Wyrwich KW. Interpretation of changes in health-related quality of life: the remarkable universality of half a standard deviation. *Med Care* 2003;41:582-92 doi:10.1097/01.MLR.0000062554.74615.4C [doi].

19 de Vet HC, Terwee CB. The minimal detectable change should not replace the minimal important difference. *J Clin Epidemiol* 2010;63:804,5; author reply 806 doi:10.1016/j.jclinepi.2009.12.015 [doi].

20 Cella D, Hahn EA, Dineen K. Meaningful change in cancer-specific quality of life scores: differences between improvement and worsening. *Qual Life Res* 2002;11:207-21.

21 Guyatt GH, Norman GR, Juniper EF, et al. A critical look at transition ratings. *J Clin Epidemiol* 2002;55:900-8 doi:S0895435602004353 [pii].

22 Guyatt GH, Jaeschke RJ. Reassessing quality-of-life instruments in the evaluation of new drugs. *Pharmacoeconomics* 1997;12:621-6 doi:10.2165/00019053-199712060-00002 [doi].

23 Wyrwich KW, Tierney WM, Wolinsky FD. Using the standard error of measurement to identify important changes on the Asthma Quality of Life Questionnaire. *Qual Life Res* 2002;11:1-7.

24 Devji T, Guyatt GH, Lytvyn L, et al. Application of minimal important differences in degenerative knee disease outcomes: a systematic review and case study to inform BMJ Rapid Recommendations. *BMJ Open* 2017;7:e015587,2016-015587 doi:10.1136/bmjopen-2016-015587 [doi].

For peer review only

25 Revicki D, Hays RD, Cella D, et al. Recommended methods for determining responsiveness and minimally important differences for patient-reported outcomes. *J Clin Epidemiol* 2008;61:102-9 doi:10.1016/j.jclinepi.2007.03.012 [doi].

26 Alma H, de Jong C, Tsiligianni I, et al. Clinically relevant differences in COPD health status: systematic review and triangulation. *Eur Respir J* 2018;52:1800412 doi:10.1183/13993003.00412.

For peer review only

INTERNATIONAL-PROSTATE SYMPTOM SCORE (IPSS)

	Not at all	Less than 1 time in 5	Less than half the time	About half the time	More than half the time	Almost always
1. Over the past 4 weeks, how often have you had a sensation of not emptying your bladder completely after you finished urinating?	0	1	2	3	4	5
2. Over the past 4 weeks, how often have you had to urinate again less than two hours after you finished urinating?	0	l	2	3	4	5
3. Over the past 4 weeks, how often have you found you stopped and started again several times when you urinated?	0	1	2	3	4	5
4. Over the past 4 weeks, how often have you found it difficult to postpone urination?	0	1	2	3	4	5
5 Over the past 4 weeks, how often has your urinary stream been weaker than usual?	0	1	2	3	4	5
6 Over the past 4 weeks, how often have you had to push or strain to begin urination?	0	1	2	3	4	5
	None	1 time	2 times	3 times	4 times	5 or more times
7. Over the past 4 weeks, how many times, in general, did you get up to urinate from the time you went to bed at night until the time you got up in the morning?	0	1	2	3	4	5

IPSS Quality of life question

	Delighted	Pleased	Mostly satisfied	Mixed - neither satisfied nor dissatisfied	Mostly dissatisfied	Unhappy	Terrible
If you were to spend the rest of your life with your urinary condition just the way it is now, how would you feel about that?	0	1	2	3	4	5	6

OAB-q-SF

This questionnaire asks about how much you have been bothered by selected bladder symptoms during the past 4 weeks. Please place a or X in the box that best describes the extent to which you were bothered by each symptom during the past 4 weeks. There are no right or wrong answers. Please be sure to answer every question.

During the past 4 weeks, how bothered were you by...

	Not at all	A little bit	Some-what	Quite a bit	A great deal	A very great deal
1. An uncomfortable urge to urinate?	☐	☐	☐	☐	☐	☐
2. A sudden urge to urinate with little or no warning?	☐	☐	☐	☐	☐	☐
3. Accidental loss of small amounts of urine?	☐	☐	☐	☐	☐	☐
4. Nighttime urination?	☐	☐	☐	☐	☐	☐
5. Waking up at night because you had to urinate?	☐	☐	☐	☐	☐	☐
6. Urine loss associated with a strong desire to urinate?	☐	☐	☐	☐	☐	☐

Patient Global Impression of Improvement, PGI-I

Have your symptoms changed since the start of the medication (the moment you completed the previous questionnaire)?

Very much better	Much better	A little better	No change	A little worse	Much worse	Very much worse
☐	☐	☐	☐	☐	☐	☐

For peer review only

STROBE Statement—Checklist of items that should be included in reports of ***cohort studies***

	Item No	Recommendation	Page No
**Title and abstract**	1	(*a*) Indicate the study's design with a commonly used term in the title or the abstract	2
		(*b*) Provide in the abstract an informative and balanced summary of what was done and what was found	2
**Introduction**			
Background/rationale	2	Explain the scientific background and rationale for the investigation being reported	4
Objectives	3	State specific objectives, including any prespecified hypotheses	4
**Methods**			
Study design	4	Present key elements of study design early in the paper	4-5
Setting	5	Describe the setting, locations, and relevant dates, including periods of recruitment, exposure, follow-up, and data collection	5
Participants	6	(*a*) Give the eligibility criteria, and the sources and methods of selection of participants. Describe methods of follow-up	5
		(*b*) For matched studies, give matching criteria and number of exposed and unexposed	
Variables	7	Clearly define all outcomes, exposures, predictors, potential confounders, and effect modifiers. Give diagnostic criteria, if applicable	5-6
Data sources/ measurement	8*	For each variable of interest, give sources of data and details of methods of assessment (measurement). Describe comparability of assessment methods if there is more than one group	5-6
Bias	9	Describe any efforts to address potential sources of bias	7
Study size	10	Explain how the study size was arrived at	-
Quantitative variables	11	Explain how quantitative variables were handled in the analyses. If applicable, describe which groupings were chosen and why	6
Statistical methods	12	(*a*) Describe all statistical methods, including those used to control for confounding	6-7
		(*b*) Describe any methods used to examine subgroups and interactions	6
		(*c*) Explain how missing data were addressed	7
		(*d*) If applicable, explain how loss to follow-up was addressed	
		(*e*) Describe any sensitivity analyses	
**Results**			
Participants	13*	(a) Report numbers of individuals at each stage of study—eg numbers potentially eligible, examined for eligibility, confirmed eligible, included in the study, completing follow-up, and analysed	7
		(b) Give reasons for non-participation at each stage	
		(c) Consider use of a flow diagram	
Descriptive data	14*	(a) Give characteristics of study participants (eg demographic, clinical, social) and information on exposures and potential confounders	8
		(b) Indicate number of participants with missing data for each variable of interest	8
		(c) Summarise follow-up time (eg, average and total amount)	8
Outcome data	15*	Report numbers of outcome events or summary measures over time	-

For peer review only - http://bmjopen.bmj.com/site/about/guidelines.xhtml

Main results	16	(*a*) Give unadjusted estimates and, if applicable, confounder-adjusted estimates and their precision (eg, 95% confidence interval). Make clear which confounders were adjusted for and why they were included	7-11
		(*b*) Report category boundaries when continuous variables were categorized	7-11
		(*c*) If relevant, consider translating estimates of relative risk into absolute risk for a meaningful time period	
Other analyses	17	Report other analyses done—eg analyses of subgroups and interactions, and sensitivity analyses	7-11
**Discussion**			
Key results	18	Summarise key results with reference to study objectives	11
Limitations	19	Discuss limitations of the study, taking into account sources of potential bias or imprecision. Discuss both direction and magnitude of any potential bias	13-14
Interpretation	20	Give a cautious overall interpretation of results considering objectives, limitations, multiplicity of analyses, results from similar studies, and other relevant evidence	11-14
Generalisability	21	Discuss the generalisability (external validity) of the study results	11
**Other information**			
Funding	22	Give the source of funding and the role of the funders for the present study and, if applicable, for the original study on which the present article is based	3

*Give information separately for exposed and unexposed groups.

Note: An Explanation and Elaboration article discusses each checklist item and gives methodological background and published examples of transparent reporting. The STROBE checklist is best used in conjunction with this article (freely available on the Web sites of PLoS Medicine at http://www.plosmedicine.org/, Annals of Internal Medicine at http://www.annals.org/, and Epidemiology at http://www.epidem.com/). Information on the STROBE Initiative is available at http://www.strobe-statement.org.

For peer review only
